# Machine learning reveals proteome-encoded growth determinants underlying metabolic versatility of *Rhodopseudomonas palustris* on lignin-derived aromatics

**DOI:** 10.1128/msystems.00383-26

**Published:** 2026-05-07

**Authors:** Abraham Osinuga, Mark Kathol, Rajib Saha

**Affiliations:** 1 Department of Chemical and Biomolecular Engineering, University of Nebraska-Lincoln 14719, Lincoln, Nebraska, USA; Danmarks Tekniske Universitet, Kgs. Lyngby, Lyngby-Taarbæk, Denmark

**Keywords:** *Rhodopseudomonas palustris*, proteomics, machine learning, growth predictions, lignin breakdown products, metabolic versatility

## Abstract

Lignin depolymerization generates mixtures of aromatic compounds that are promising carbon sources for microbial bioconversion, yet the constraints governing microbial growth on these substrates remain unclear. Here, we investigated how *Rhodopseudomonas palustris* CGA009 organizes metabolism to grow on chemically distinct lignin-derived aromatics under aerobic and anaerobic conditions. Quantitative proteomics across 14 substrate-oxygen environments revealed extensive oxygen-dependent proteome remodeling, consistent with shifts between respiratory and photoheterotrophic programs. Despite this reorganization, predictive modeling showed that growth rate is encoded by a relatively small subset of proteins whose abundance tracks physiological performance across environments. Using CorePredX, a machine-learning framework that combines global importance analysis with dependence-aware redundancy filtering, we identified 118 high-confidence proteins whose variation made conditionally non-redundant contributions to growth-rate prediction among 1,857 quantified proteins. These proteins are organized into a hierarchical architecture comprising a cross-condition core, adaptive regulators, substrate-specific specialists, and conditional hubs. The cross-condition core linked translational capacity, sulfur assimilation, redox buffering, and carbon storage cycling, highlighting conserved proteomic features associated with growth across oxygen regimes and substrate chemistries. Notably, an uncharacterized cystathionine beta-synthase domain protein, RPA3416, emerged as a strong predictive component of this core, raising the hypothesis of an adenylate-responsive process associated with aerobic growth on lignin-derived aromatics. Together, these results suggest that the metabolic versatility of *R. palustris* arises from flexible regulation layered onto partially conserved proteome-encoded growth-associated constraints, providing candidate targets for lignin bioconversion engineering and metabolic model refinement.

**IMPORTANCE:**

Lignin is an abundant part of plant waste that is notoriously difficult to break down and turn into valuable products. While certain microbes, like *Rhodopseudomonas palustris*, can consume lignin byproducts, scientists do not fully understand how they do it. In this study, we discovered that we can predict how well this microbe will grow simply by tracking a small, specific group of its core proteins—even when its environment changes. We also identified a new, unstudied protein that is a strong candidate for future research. By pinpointing the specific biological tools microbes use to process lignin, this research provides a clear roadmap for engineering bacteria to efficiently turn plant waste into useful, sustainable materials.

## INTRODUCTION

Lignin depolymerization releases a chemically heterogeneous pool of aromatic compounds that vary widely in redox state, energy yield, and catabolic accessibility—properties that translate directly into differences in the energetic cost and metabolic complexity of their assimilation (1, 2). *Rhodopseudomonas palustris*, a metabolically versatile phototrophic α-proteobacterium, is among the few organisms capable of catabolizing many of these compounds under both aerobic and anaerobic conditions, switching between respiratory and photoheterotrophic growth programs as oxygen availability dictates (3–5). We previously characterized the underlying catabolic mechanisms using a multi-omics approach, proposing a promiscuous p-coumarate (pC)-anchored superpathway funneling H-, G-, and S-type lignin units toward the TCA cycle and demonstrating that ligninolysis is constrained primarily by NAD^+^ availability rather than catabolic enzyme abundance (6). However, growth on lignin-derived aromatics is highly sensitive to substrate chemistry and redox regime: closely related compounds support markedly different growth rates, some require co-substrate supplementation to sustain catabolism, and methoxylated monolignols resist assimilation entirely. This phenotypic heterogeneity is not explained by pathway presence alone—*R*. *palustris* encodes the enzymatic machinery for aerobic β-ketoadipate funneling, anaerobic benzoyl-CoA reduction, and peripheral demethylation—suggesting instead that growth limitation reflects higher-order constraints on proteome allocation, redox balance, and metabolic coordination that vary with substrate chemistry and oxygen availability (7, 8).

Understanding these constraints matters for both fundamental and applied reasons. Mechanistically, metabolic versatility in facultative phototrophs has long been attributed to the flexibility of their regulatory networks, but it remains unclear whether this flexibility operates through wholesale rewiring of growth control across environments or through condition-specific deployment of a conserved biochemical scaffold. Practically, identifying the candidate proteomic bottlenecks associated with growth on lignin-derived carbon is a prerequisite for rational engineering of microbial lignin valorization—a process whose efficiency depends on understanding and relieving the biochemical constraints that restrict growth rate and aromatic carbon assimilation across chemically diverse substrate mixtures, constraints that cannot be inferred from pathway composition alone.

Existing approaches have made important progress but face systematic limitations. Global transcriptomic and proteomic studies reveal extensive pathway induction and repression during growth on individual lignin-derived substrates; however, they typically identify hundreds of responsive proteins without clarifying which changes are functionally necessary for growth across environments (9, 10). Recent machine-learning models trained on *R. palustris* transcriptomic and proteomic data improve on differential-abundance analyses by directly linking omics features to growth-rate prediction and identifying a compact set of transport and signaling proteins associated with lignin catabolism (11). However, feature-importance scores derived within a fixed condition set do not establish whether these proteins remain predictively necessary across chemically and redox-diverse environments, or whether their apparent importance is nonredundant with correlated proteins. Functional redundancy compounds this problem: multiple transporters, oxidoreductases, and regulators can substitute for one another (6), so differential abundance or co-expression does not establish predictive necessity. Weighted gene co-expression network analysis (WGCNA) and related methods identify modules of co-abundant proteins whose collective trend correlates with growth rate, but correlation with growth does not by itself establish whether those proteins uniquely inform growth prediction across environments—proteins that rise and fall with biomass accumulation as passive participants in a coordinated response are indistinguishable from more directly informative features by network methods alone (12). Genome-scale metabolic (M) and metabolic expression (ME) models have advanced mechanistic understanding of substrate- and energy-dependent trade-offs in *R. palustris*, including redox partitioning and electron allocation between carbon and nitrogen fixation (10, 13), but are architecturally constrained to metabolic enzymes and cannot readily incorporate the transporters, regulators, and stress-response factors that quantitatively influence growth in ways not captured by stoichiometric flux balance.

What is needed is a framework that asks a different question: not which proteins change most between conditions, but which proteins a generalizing model must rely upon to accurately predict how fast the organism grows across chemically and redox-diverse environments—and whether that reliance remains conditionally non-redundant after accounting for correlated proxies. Here, we address this by treating the quantitative proteome as an integrative readout of cellular state and asking whether growth-rate variation across lignin breakdown products (LBPs) is predictable from proteomic composition, which proteomic features drive that prediction, and whether those features retain predictive influence after dependence-aware filtering. We introduce CorePredX, a cross-condition neural modeling and interpretive framework that couples supervised growth-rate prediction with Monte Carlo SHAP attribution and a two-stage dependence-aware redundancy analysis to identify proteins whose quantitative variation encodes growth potential across environments. Applied to *R. palustris* CGA009 grown on a panel of lignin-derived aromatics under aerobic and anaerobic conditions, CorePredX resolves a compact four-tier hierarchy of candidate growth-associated proteins that extends beyond what differential abundance or co-expression analysis recovers, outlines a conserved cross-condition predictive scaffold, and prioritizes a previously uncharacterized cystathionine beta-synthase (CBS)-domain protein as a leading aerobic growth-associated candidate—supporting the hypothesis that metabolic versatility in this organism may arise less from fully distinct growth programs than from flexible regulatory access to partially shared proteome-encoded constraints.

## RESULTS

### *R. palustris* grows on lignin breakdown products

**Fig 1:**
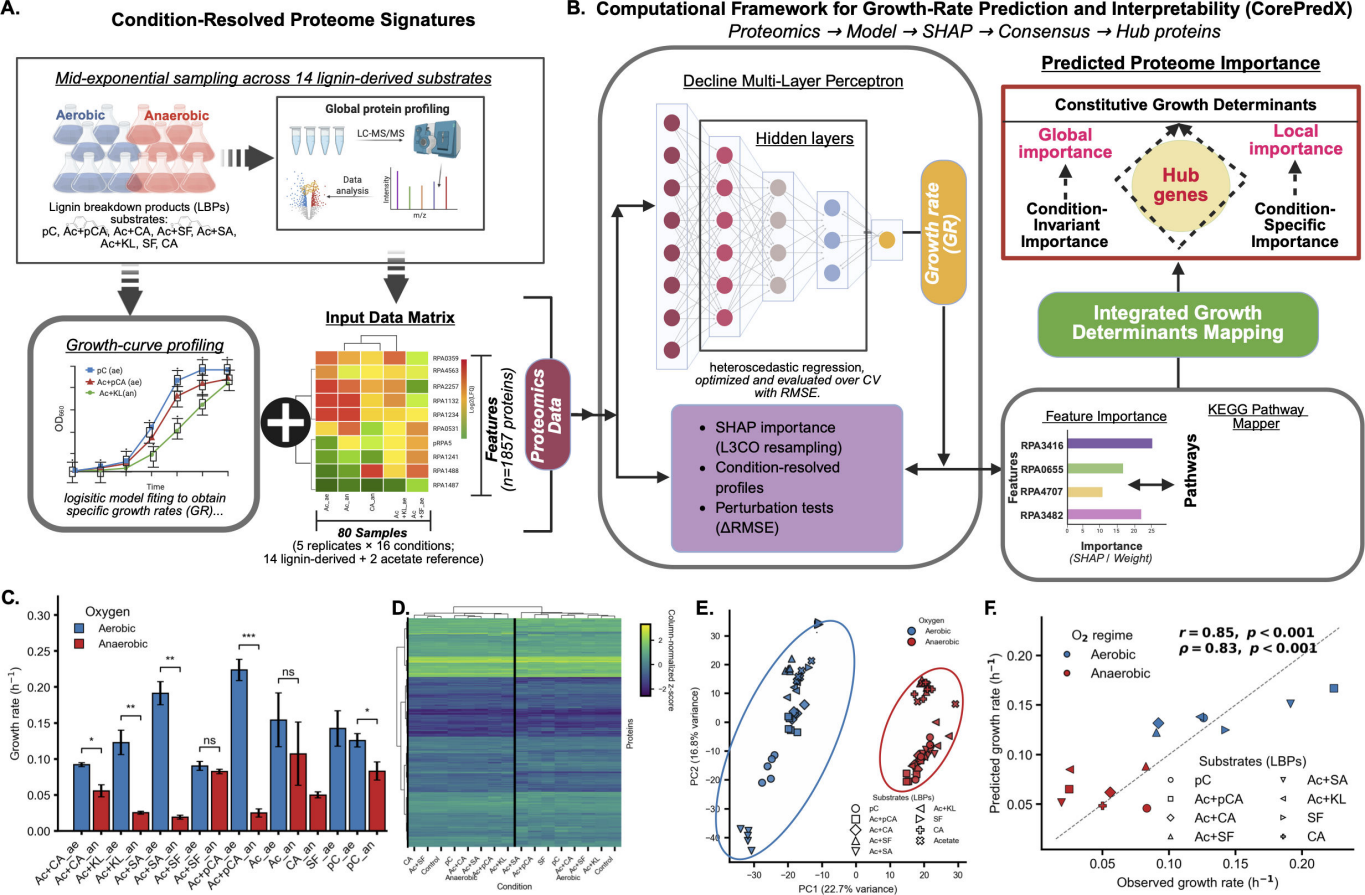
Experimental and computational framework of CorePredX. (**A**) *R*. *palustris* was grown across 16 lignin-derived substrates under aerobic or anaerobic conditions, yielding condition-resolved proteomes (1,857 proteins). (**B**) CorePredX trains a tapered multilayer perceptron (MLP) with condition-anchored cross-validation and Monte-Carlo SHAP to obtain global and condition-specific feature contributions, culminating in a refined hub set. (**C–F**) Growth-rate responses, proteome clustering, PCA separation of oxygen regimes, and high observed–predicted agreement demonstrate the biological structure captured by CorePredX. In panel **C**, statistical significance is denoted as *P* < 0.05 (*), *P* < 0.01 (**), and *P* < 0.001 (***); ns, not significant.

We first quantified the capacity of *R. palustris* to grow on a panel of LBPs relevant to kraft lignin (KL) depolymerization. Cultures were supplied with pC, p-coumaryl alcohol (pCA), coniferyl alcohol, sinapyl alcohol, sodium ferulate, or KL as potential carbon sources, with acetate (Ac) retained as a reference substrate because prior work showed that only a subset of LBPs—most notably p-coumarate—could support growth as sole carbon sources, whereas more recalcitrant substrates required acetate co-feeding, consistent with a higher energetic and catabolic burden of assimilation (6, 14) (Fig. 1A). Acetate is widely recognized as a privileged carbon and electron source for *R. palustris*, supporting robust photoheterotrophic growth via direct assimilation into central metabolism and efficient redox balancing, in contrast to the more energetically or physiologically constrained utilization of many lignin-derived aromatics (15–19). Growth curves were generated under aerobic and anaerobic conditions and fitted with logistic models (Fig. S1; Table S1), yielding condition-specific growth-rate parameters (Fig. 1A and C) that define the phenotypic space for subsequent predictive analyses. Across conditions, growth rate and maximum biomass density exhibited a pronounced inverse association, with faster-growing cultures tending to reach lower final biomass densities (Fig. S1C through E). This relationship was evident under aerobic conditions but absent under anaerobic growth, suggesting that oxygen availability permits alternative metabolic strategies that balance rapid energy generation against biomass yield. These measurements define the phenotypic landscape associated with chemically distinct lignin-derived substrates across redox regimes.

Consistent with prior studies of lignin-derived aromatic utilization, several substrates failed to support sustained growth as sole carbon sources under either redox regime (4, 5). For these conditions, supplementation with acetate was required to supply ATP and reducing equivalents necessary to initiate peripheral aromatic catabolism. Importantly, acetate alone does not engage the aromatic degradation pathways encoded by *R. palustris*, as these genes are transcriptionally repressed or non-induced in the absence of an aromatic substrate, regardless of oxygen availability (20, 21). To ensure that lignin breakdown products contributed actively to biomass formation rather than serving as inert co-substrates, cultures supplemented with acetate were required to exceed the final optical density of acetate-only controls. Under this criterion, *p*-coumarate uniquely supported robust and reproducible growth without acetate under both aerobic and anaerobic conditions, whereas sodium ferulate and coniferyl alcohol supported more limited, condition-dependent growth in the absence of acetate, consistent with higher energetic or redox constraints on their assimilation (6, 9). In contrast, methoxylated monolignols and kraft lignin remained refractory, delineating substrate-specific energetic thresholds that constrain lignin-derived carbon assimilation in *R. palustris*.

### Oxygen availability reorganizes central metabolism and photosynthetic machinery

Because anaerobic growth in *R. palustris* requires engagement of a photoheterotrophic metabolic program, we verified that anoxic cultures exhibited the expected physiological and proteomic signatures before proceeding to quantitative proteomics. Under anaerobic conditions, proteins associated with the photosynthetic reaction center and bacteriochlorophyll biosynthesis were strongly induced—including light-harvesting complex proteins *pucBA* (RPA2654) and *pucBC* (RPA3009), reaction-center subunits *pufL* (RPA1527) and *pufM* (RPA1528), bacteriochlorophyll biosynthesis enzymes *bchD* (RPA1507), *bchP* (RPA1532), *bchM* (RPA1546), and *bchE* (RPA1668), and carotenoid biosynthesis enzymes *crtI* (RPA1512), *crtB* (RPA1513), and *crtE* (RPA1519)—supporting light-driven electron flow and ATP generation via cyclic photophosphorylation (Fig. S1F). This transition was accompanied by reciprocal regulation of porphyrin biosynthesis, with repression of the oxygen-dependent coproporphyrinogen oxidase *HemF* (RPA1514) and induction of oxygen-independent isoforms *HemN1* (RPA1666) and *HemN2* (RPA0327), alongside reduced abundance of cytochrome aa3 oxidase components such as *CoxB* (RPA0831). Aromatic catabolism exhibited a correspondingly structured switch: aerobic conditions favored expression of β-ketoadipate pathway enzymes *pcaF* (RPA0513) and *pcaC* (RPA4740), whereas anaerobic conditions selectively induced the benzoyl-CoA pathway regulator *badR* (RPA0655) and associated enzymes *badE* (RPA0658), *badD* (RPA0654), and *badI* (RPA0653). This system-wide remodeling was reflected globally in the proteome, with anaerobic conditions forming a coherent block in the clustered heatmap (Fig. 1C), separating cleanly from aerobic samples in PCA space (Fig. 1E) and across the full quantified proteome (Fig. S3A). These findings indicate a strong activation of photosynthetic machinery and related pathways under anoxic, light-exposed conditions, consistent with *R. palustris* physiology.

### Quantitative proteome states encode growth rate across lignin-derived substrates

With growth phenotypes and oxygen-dependent metabolic identity established, we asked a more direct question: do quantitative proteome states contain sufficient information to predict how fast *R. palustris* grows on chemically distinct lignin breakdown products? Five biological replicates were collected per substrate-oxygen condition using label-free quantitative (LFQ) proteomics, sampled at mid-exponential phase—the point of maximal instantaneous growth inferred from logistic fits (Fig. 1A and C)—to capture the metabolic and regulatory state actively sustaining biomass accumulation while minimizing confounding effects of stationary-phase stress or substrate depletion (Fig. 1C and D). The resulting profiles—comprising 80 samples (5 biological replicates across 16 substrate-oxygen conditions: 14 lignin-derived conditions plus 2 acetate reference conditions; 8 substrates × 2 oxygen regimes) and quantifying 1,857 proteins—were used as input to CorePredX, a supervised machine-learning framework that maps high-dimensional protein abundance states to physiological growth outcomes across environmental contexts (Fig. 1B). Rather than asking which proteins change the most, CorePredX asks which proteins collectively encode observed variation in growth rate, combining a predictive model with *post-hoc* Shapley additive explanation (SHAP) attribution to decompose condition-level growth-rate determinants (Materials and Methods; Method S1). Model performance was evaluated using a condition-anchored leave-one-condition-out cross-validation (LOOCV) strategy in which all five replicates of a held-out condition were excluded from training while acetate references were retained in every fold, providing a stable aerobic and anaerobic anchor states for calibration while also making the evaluation partly dependent on those anchor conditions.

Across all folds, predicted growth rates closely tracked measured values (Fig. 1F; root mean square error [RMSE] = 0.028 ± 0.018 h⁻¹), corresponding to ~13%–15% of the observed physiological range (0.02–0.22 h⁻¹). Condition-averaged predictions preserved both absolute magnitudes and rank ordering across substrates and oxygen regimes (Pearson *r* = 0.85, Spearman ρ = 0.83; both *P* < 0.001), indicating that growth-rate variation across lignin-derived substrates is encoded in structured, reproducible features of the proteome rather than solely in idiosyncratic condition-specific effects. This predictive sufficiency indicates that reproducible proteomic features encode growth-rate variation across environments and motivates downstream analysis of candidate growth-associated processes.

### Growth-predictive proteins separate into regime-invariant features and condition-adaptive modulators

**Fig 2:**
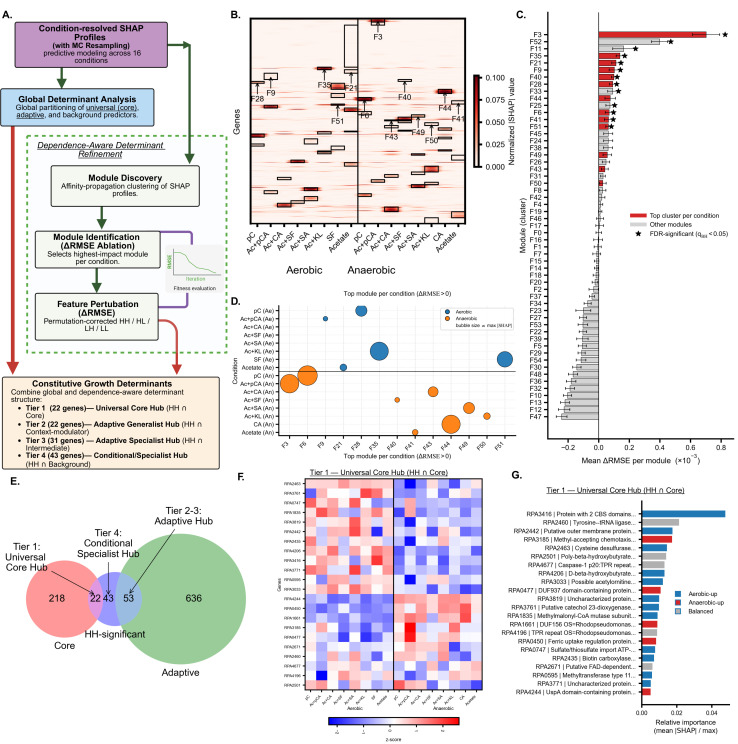
Modular and dependence-aware analysis identifies universal and context-linked growth determinants in *R. palustris*. (**A**) Integrated framework combining global determinant analysis with SHAP-derived module discovery, module ablation, and dependence-aware feature perturbation. (**B**) Condition-resolved SHAP profiles reveal modules with aerobic–anaerobic shifts in predictive influence. (**C**) Module ablation (ΔRMSE) identifies statistically significant predictive modules (false discovery rate [FDR] < 0.05). (**D**) Highest-impact module per condition highlights substrate- and oxygen-specific reliance patterns. (**E**) Four-tier hierarchy of constitutive growth determinants: tier 1 universal core, tier 2 adaptive generalists, tier 3 adaptive specialists, and tier 4 conditional specialists. (**F**) Tier 1 universal core hub: proteomic abundance profiles showing regime-invariant expression patterns, consistent with their universal predictive role. (**G**) Corresponding relative SHAP importance of the 22 high-confidence core members.

Although accurate growth prediction establishes that growth-rate variation is encoded in the proteome, it does not by itself reveal which components most consistently inform growth prediction across environments. To resolve this, we applied a global determinant analysis that evaluates the persistence of model reliance across conditions using SHAP-derived (22) predictive contributions (Fig. 1B; Fig. 2A; Materials and Methods; Method S2), assessing consistency of feature importance rather than invariance in protein abundance or direct biochemical causality. Critically, this analysis operates entirely in feature-importance space—assessing how reliably the model depends on each protein across environments—rather than in abundance space and therefore does not assume or require invariant protein expression. This approach identified a universal core determinant set of 240 proteins exhibiting high global importance with minimal oxygen-associated variance, indicating that the model repeatedly relies on these features when predicting growth across conditions. In Method S2, high-importance proteins were defined as those above the 75th percentile of global mean SHAP importance, and regime-associated effects were partitioned using regime-variance fractions and Welch tests with Benjamini-Hochberg correction. In contrast, most proteins fell into diffuse background intermediate predictors (7, 8, 10, 23) (928 proteins) or adaptive modulators whose predictive contributions shifted with substrate or oxygen context (225 and 464 proteins). Notably, after multiple-testing correction, no proteins exhibited predictive influence statistically exclusive to either aerobic or anaerobic conditions—a result that does not contradict the strong oxygen-driven bifurcation in expression space (Fig. 1E), but instead highlights the model’s capacity to identify shared growth-associated predictive features that persist across divergent expression programs.

### Growth-predictive information is concentrated in a compact set of non-redundant proteomic determinants

The global determinant analysis ranks proteins by the consistency of the model’s reliance on them across conditions, but high mean feature importance (SHAP based) alone does not confirm that a protein’s contribution is conditionally non-redundant in the predictive model. A protein can rank as a persistent determinant simply because its abundance co-varies with a more directly informative growth-associated feature—the model uses it as a correlated proxy, and removing it would have little effect on prediction because the information it carries is already encoded elsewhere in the proteome. To identify proteins whose quantitative variation is uniquely required for accurate prediction after accounting for correlated structure, we implemented a two-stage dependence-aware high-confidence determinant (HH) analysis (Materials and Methods; Methods S3 to S6).

In the first stage, we organized proteins into coherent analytical units by clustering their condition-resolved SHAP profiles using affinity propagation, yielding 55 proteome-wide modules that capture distinct patterns of model reliance across environments (Method S4; Fig. 2A through C). Importantly, sharing a module does not imply shared function—it means the model draws on these proteins in similar ways across conditions. We then tested each module’s necessity by systematically permuting its abundance values while conditioning on the rest of the proteome and measuring the resulting increase in prediction error across all 364 Monte Carlo cross-validation iterations (Method S5; Fig. 2C). Only 13 of 55 modules produced a statistically significant degradation in performance upon perturbation (false discovery rate [FDR] < 0.05), and selecting the highest-impact module per substrate–oxygen condition confirmed that these 13 modules collectively account for the model’s predictive dependence across all environments (Fig. 2D).

In the second stage, we resolved which individual proteins within those 13 modules carry conditionally non-redundant influence by applying an analogous feature-level conditional perturbation test—permuting each protein’s residual abundance after conditioning on all others, both within its module and across the full proteome, and comparing the resulting effect sizes under both conditioning definitions (Method S6). Proteins passing both statistical and effect-size thresholds under both conditioning regimes were designated HH (BH-corrected *q* < 0.05). This dual-conditioning criterion specifically identifies proteins whose predictive contribution persists despite collinearity, pathway coupling, or shared regulatory structure, distinguishing non-redundant predictive features from redundancy-mediated effects. Applying these criteria identified 118 high-confidence determinants whose perturbation consistently degraded model performance across lignin carbon sources and oxygen regimes (Fig. 2A; Fig. S2 and S3). Fewer than 7% of quantified proteins meet this threshold, indicating that although growth-rate-associated predictive signal in *R. palustris* spans multiple biological processes, it is concentrated within a narrowly defined, non-redundant feature set. This is notably more compact than the broad proteomic sectors identified as growth-limiting in *E. coli* (24), or the hundreds of correlated transcripts recovered by gene-signature approaches in yeast (25), consistent with emerging sparse-modularity principles suggesting that while thousands of proteins respond to changes in growth rate, a much smaller subset may dominate cross-condition predictive signal (23). Crucially, this layer refines rather than recapitulates the global determinants: many broadly influential proteins fail the HH criterion due to redundancy, while several moderate contributors prove unexpectedly persistent under conditional perturbation, establishing a precisely bounded protein set for mechanistic follow-up. Whether this computational non-redundancy reflects biological non-substitutability remains unresolved without experimental perturbation.

### A hierarchical organization of growth determinants emerges from proteome-wide analysis

Despite the pronounced metabolic versatility of *Rhodopseudomonas palustris*, the oxygen-driven bifurcation of its proteome (Fig. 1E; Fig. S3A) reflects a well-established role of oxygen as a primary organizer of bacterial proteome allocation and metabolic state (26, 27), but this separation alone does not identify which proteins most consistently inform growth prediction across environments. By integrating global determinant analysis with the dependence-aware HH framework—both defined in the space of SHAP-derived model reliance rather than protein abundance—we resolved a compact, four-tier hierarchy of growth-relevant proteins that extends beyond correlative importance (Fig. 2A). The universal core hub (tier 1) is highly constrained, comprising only 22 high-confidence, regime-invariant proteins, in contrast to the 96 model-designated non-redundant adaptive modulators distributed across tiers 2–4 (Fig. 2E). To place this hierarchy in functional context, all 118 HH-significant proteins were annotated against Kyoto Encyclopedia of Genes and Genomes, classifying proteins as directly metabolic only when catalyzing registered reactions; consistent with the non-model status of *R. palustris* CGA009, many annotations remain putative. Only 42 proteins (36%) mapped to known metabolic pathways, whereas the majority (64%) comprised proteins of putative function, transcriptional regulation, or environmental sensing (Table S4). This functional partition is reinforced by condition-resolved SHAP signatures (Fig. 2B through D), by the mixed oxygen-regime dependence defined by statistically significant differential abundance in the proteomic data (Fig. 2F and G), and by the persistence of structured—but attenuated—aerobic-anaerobic organization when analysis is restricted to the HH-significant subset (Fig. S3B).

Across the four-tier hierarchy, mapped metabolic proteins are not uniformly distributed. Although the universal core hub (tier 1) contains a substantial fraction of metabolic enzymes (8 of 22; 36%), tier 2 adaptive generalists hub is comparatively enriched for non-metabolic or putative regulators (6 of 22 metabolic; 27%), consistent with broad, context-dependent modulation of core pathways rather than direct catalytic control. Tier 3 adaptive specialists (31 proteins) exhibit intermediate global importance with growth-predictive importance/influence confined to specific lignin substrates or oxygen regimes, reflecting localized enzymatic or regulatory functions, whereas tier 4 conditional/specialist hubs (43 proteins) display the highest metabolic density (17 of 43; 40%) yet contribute predictive power only under narrowly defined environmental conditions. This organization delineates a minimal, regime-spanning predictive backbone associated with growth across environments, overlaid by progressively specialized proteins that tune growth to substrate chemistry and redox state. Importantly, predictive influence remains distributed across a small set of high-leverage features rather than collapsing onto a single dominant protein (Fig. S2), indicating that growth emerges from multiple partially redundant biochemical constraints rather than reliance on a single condition-linked proxy.

### A candidate cross-condition growth core links biosynthetic capacity, redox balance, and carbon assimilation

Integrating quantitative proteomic abundance patterns with the tiered hierarchy and condition-resolved model structure (Fig. 2B; Fig. S3C through F) reveals growth control as a layered process rather than a diffuse property of the oxygen-partitioned proteome. Within the universal core hub, several proteins maintain stable predictive influence across oxygen regimes while remaining model-designated non-redundant determinants associated with growth. These include tyrosyl-tRNA synthetase TyrS (RPA2460), reflecting sustained translational capacity, and poly-β-hydroxybutyrate synthase PhbC (RPA2501), which governs carbon storage and mobilization under fluctuating nutrient availability (28, 29). Complementing PhbC, D-3-hydroxybutyrate dehydrogenase (RPA4206) catalyzes the interconversion of D-3-hydroxybutyrate and acetoacetate, enabling PHB-derived carbon to re-enter central metabolism as acetyl-CoA (28). The regime-invariant co-occurrence of both PhbC and RPA4206 within the universal core suggests that PHB synthesis and mobilization may constitute a coupled axis associated with growth variation, consistent with the established role of PHB as a redox and carbon buffer in purple non-sulfur bacteria under fluctuating substrate availability (30).

Other core members exhibit oxygen-associated abundance shifts yet retain regime-invariant predictive influence, indicating redistribution of conserved biochemical functions rather than substitution of growth-associated predictive structure. These include cysteine desulfurase NifS1 (RPA2463), which supports Fe-S cluster assembly and thiamine biosynthesis (31); biotin carboxylase AccC (RPA2435), central to carboxylation reactions in fatty-acid and anaplerotic metabolism (32); methylmalonyl-CoA mutase MutB (RPA1835), which links propionate and branched-chain metabolism to central carbon flux (33); and iron- and stress-responsive regulators including Fur (RPA0450) and the UspA-like protein RPA4244, which coordinates iron homeostasis and stress adaptation under redox limitation (34, 35). Reinforcing the sulfur assimilation node, the sulfate/thiosulfate import ATPase CysA (RPA0747) imposes a complementary constraint at the point of inorganic sulfur uptake. CysA is the catalytic subunit of the CysATW ABC-type transporter, which constitutes the primary route for sulfate and thiosulfate entry in diverse bacteria; deletion of cysA produces cysteine auxotrophy, establishing non-redundant essentiality for sulfur assimilation (36). Imported sulfate feeds cysteine biosynthesis, which supplies precursors for protein synthesis, glutathione, and the Fe-S cluster assembly reaction catalyzed by NifS1 (37). The co-occurrence of CysA and NifS1 within the same regime-invariant universal core, therefore, suggests a coherent sulfur-assimilation node whose predictive non-redundancy may reflect the low availability of organic sulfur in the minimal photosynthetic medium used here.

The core further includes an FAD-dependent monooxygenase (RPA2671) whose abundance is invariant across oxygen regimes (Fig. 2F), linking cofactor biosynthesis to universal growth capacity, and a putative catechol 2,3-dioxygenase (RPA3761), whose pattern is consistent with a possible role in aerobic lignin-derived aromatic degradation across substrates. Moreover, the methyl-accepting chemotaxis receptor MCP (RPA3185) adds a sensory candidate to the universal core. MCPs are the primary environmental sensors in bacteria and archaea, coupling extracellular ligand recognition to intracellular signaling through the CheA-CheY phosphorelay that governs directional motility (38). In metabolically versatile proteobacteria, MCPs have been shown to directly sense aromatic acids and organic carbon sources. The mechanistic basis for MCP (39) RPA3185 as a regime-invariant determinant is unclear, given the uniform liquid culture conditions used here; potential roles in signal transduction independent of directional motility, or indirect co-regulation with substrate-sensing regulons, remain to be established experimentally. In addition, acetylornithinase ArgE (RPA3033) catalyzes the penultimate deacetylation step in *de novo* L-arginine biosynthesis (40). Arginine is an immediate precursor for polyamine biosynthesis, and polyamine pools are required for ribosome biogenesis, translation fidelity, and cell growth across bacteria (41). Its regime-invariant predictive contribution therefore connects nitrogen-assimilatory sufficiency to translational capacity—a linkage consistent with established bacterial growth-law frameworks in which amino acid biosynthetic throughput is associated with growth rate (24).

Additionally, among the universal core hub members, the protein with the highest predictive importance under aerobic conditions is RPA3416, an uncharacterized protein bearing two tandem CBS domains (Fig. 2G). CBS domains are highly conserved regulatory modules found across all domains of life; in prokaryotes, they function primarily as adenosine-nucleotide-binding sensors—responding to AMP, ADP, and ATP—and translate cellular energy charge into conformational changes that regulate the activity of the hosting protein or its binding partners (42, 43). Standalone CBS-domain proteins, which consist of little else beyond the paired CBS fold, are disproportionately represented in bacteria with complex metabolic lifestyles, yet their physiological roles remain poorly understood (43). The best-characterized bacterial instances are CBS domain-containing family II inorganic pyrophosphatases (CBS-PPases), in which AMP and ADP inhibit enzymatic activity while ATP activates it, directly coupling pyrophosphate flux to the adenylate energy charge of the cell (44). The aerobic-dominant, regime-consistent predictive importance of RPA3416 therefore raises the testable hypothesis that it may function as an adenylate-responsive regulator affecting biosynthetic output during respiration on lignin-derived substrates—a condition under which high respiratory flux may generate substantial adenylate dynamics. Experimental validation through nucleotide-binding assays, co-immunoprecipitation, and deletion-strain growth phenotyping will be required to confirm causality. Collectively, these features outline a candidate conserved biochemical scaffold associated with growth variation, even as expression programs diverge across oxygen regimes.

### Adaptive regulatory and redox modules tune growth across substrate chemistry and oxygen availability

The second tier appears to capture part of the regulatory interface through which oxygen availability reshapes growth-associated predictive structure without fully redefining the core scaffold. Rather than representing direct catalytic bottlenecks, proteins in this tier appear to modulate access to substrates, redox balance, and biosynthetic throughput within an otherwise conserved backbone. Accordingly, tier 2 is enriched for transcriptional regulators, transport systems, and redox-balancing functions and aligns closely with condition-specific SHAP growth predictive modules that dominate model reliance across environments (Fig. 2B; Fig. S2B). Notably, the transcriptional regulator badR (RPA0655) emerges as a prominent hub protein—experimentally characterized as a regulator of anaerobic benzoate and aromatic acid metabolism in *R. palustris*, acting upstream of the benzoyl-CoA pathway under photoheterotrophic conditions (3, 9, 45–47). Additional regulators—including a GntR-family transcriptional regulator (RPA2343), alongside transport proteins such as an OprF-like porin (RPA4678) and a branched-chain amino acid ABC transporter ATPase (RPA1791)—indicate that growth modulation under oxygen limitation is governed primarily by regulatory gating of substrate access rather than large-scale replacement of core metabolic constraints (48). The 4-carboxymuconolactone decarboxylase PcaC (RPA4740) within this tier provides a direct biochemical anchor to oxygen-dependent β-ketoadipate pathway activity—a canonical route for aerobic aromatic funneling (49, 50)—while ferredoxin-NADP^+^ reductase Fpr (RPA1578), argininosuccinate synthase ArgG (RPA0392), acetolactate synthase IlvB (RPA3763), and ribosomal protein RpsD (RPA1589) collectively link translational capacity, redox poise, and amino acid biosynthesis to adaptive growth modulation (24, 51, 52).

Another element of adaptive control is the 5′-nucleotidase SurE (RPA2841), known to regulate nucleotide-pool homeostasis by hydrolyzing ribo- and deoxyribonucleoside monophosphates (53). By recycling nucleotides and preventing accumulation of inhibitory intermediates during metabolic transitions, SurE is plausibly linked to purine and pyrimidine homeostasis required for optimal growth (53, 54). Thus, SurE may reduce the energetic burden of *de novo* nucleotide synthesis and support nucleic-acid production under growth-constraining conditions. The Sec translocon accessory subunit YajC (RPA2832) extends the transport theme to protein secretion itself: as a component of the SecYEG-YajC-YidC membrane protein insertase supercomplex, YajC facilitates integration of membrane proteins and translocation of periplasmic proteins (55), directly conditioning the functional capacity of the membrane-localized transporters and cytochrome-containing oxygenases required for aromatic catabolism. The flavin-dependent monooxygenase RPA3700, a bacterial luciferase homolog, is consistent with a broad cofactor-recycling function: in aromatic catabolism, flavin-recycling capacity is critical because ring-cleavage dioxygenases and Baeyer-Villiger monooxygenases require a continuous supply of reduced flavin to sustain oxidative funneling (56). RPA3541, carrying a VbhA domain that modulates VirB/T4SS-type machineries in Alphaproteobacteria (57), suggests context-dependent regulation of extracellular signaling or substrate sensing, while RPA1624, annotated as a YHS domain protein proposed to sense oxidative stress or heavy metal ions (58), adds a context-dependent redox or metal-sensing candidate. Collectively, these patterns indicate that adaptation between aerobic and anaerobic states recruits oxygen-enabled aromatic cleavage capacity and regulates CoA-centered catabolism, while emphasizing regulatory gating of substrate access and redox scaffolding as the primary adaptive levers—an interpretation consistent with established *R. palustris* physiology while elevating a broad set of regulatory and accessory proteins as mechanistic candidates.

### Substrate- and regime-specific predictive features are encoded in the lower tiers

The most localized, substrate-contingent biology is concentrated in tiers 3 and 4, where specialized enzymatic and regulatory modules are recruited only when particular metabolic routes, redox chemistries, or substrate entry points are engaged. These proteins do not exert universal influence in the model, but instead become informative under defined environmental demands. In tier 3, proteins associated with oxidative metabolism and central carbon flux—including an NADH:ubiquinone oxidoreductase subunit (RPA2421), dihydrolipoyllysine succinyltransferase SucB (RPA0188), anthranilate synthase amidase TrpG (RPA4498), and a putative long-chain fatty-acid-CoA ligase (RPA2714)—are preferentially associated with oxygen-enabled metabolic states, consistent with established coupling between respiration, aromatic amino-acid biosynthesis, and growth (59–61). The non-homologous end joining protein Ku (RPA3651) introduces a DNA-repair candidate specific to aerobic conditions, consistent with elevated double-strand break frequency from reactive oxygen species generated during respiratory aromatic catabolism (62). Phasin RPA3770 adds a substrate- and regime-specific carbon-flux candidate: under anaerobic photoheterotrophic conditions, PHB granule formation and mobilization serve as primary electron sinks when photosynthetic electron flow decouples from biosynthetic demand (63). In contrast, proteins linked to iron acquisition (FbpA; RPA4152), protein maturation (peptide deformylase Def; RPA0621), and redox-sensitive dehydrogenase activity (RPA1853) dominate under oxygen-limited conditions, reflecting resource acquisition and environmental sensing strategies when oxidative chemistries are unavailable (38, 64, 65). Ribonuclease R (RPA3125), acyl-CoA dehydrogenase (RPA1612), morphinone reductase (RPA4403), and N-carbamoyl-beta-alanine amidohydrolase (RPA1745) each introduce additional substrate-specific associations with RNA surveillance, beta-oxidation-linked aromatic intermediate processing, enone detoxification, and CoA precursor supply, respectively (66–71)—further illustrating that substrate catabolism places demand on auxiliary systems not captured by pathway-centric models.

In the most conditional tier 4, the cell cycle master regulator CtrA (RPA1632) emerges as a context-linked candidate specifically under substrate-dependent conditions. CtrA is an essential response regulator in alphaproteobacteria that controls cell cycle progression and directly silences the chromosomal origin of replication during appropriate growth phases; its tier four classification is consistent with substrate-dependent modulation of cell cycle timing, suggesting that nutrient quality from different lignin-derived (72–74) substrates may differentially influence cell division throughput. The PII nitrogen regulatory protein GlnB (RPA2966) connects carbon-nitrogen status sensing to growth-rate control: PII proteins sense the intracellular C/N balance through allosteric responses to 2-oxoglutarate, ATP/ADP, and glutamine (75), and in purple non-sulfur bacteria closely related to *R. palustris*, GlnB homologs regulate nitrogen fixation, glutamine synthetase adenylylation, and energy-status sensing (76, 77). Glutathione reductase Gor (RPA1983) and glutathione S-transferase Gst2 (RPA0820) define a glutathione-dependent redox-buffering system with a substrate-specific demand likely to be most acute during high-flux aerobic aromatic degradation that generates quinone intermediates and reactive electrophilic products (78, 79). The ribosome hibernation-promoting factor HPF (RPA0051) adds ribosome protection as a conditionally engaged candidate: HPF-mediated 100S ribosome dimer formation sequesters translational capacity during nutrient fluctuation and prevents irreversible ribosome degradation during recovery (80, 81)—a plausible constraint under anaerobic conditions where energy supply from photophosphorylation may oscillate across substrates. Vanillate O-demethylase VanA (RPA3619) and acyl-CoA synthetase RPA2302 anchor tier 4 to lignin-aromatic entry and CoA-ester activation, respectively, representing the most substrate-specific biochemical access points in the hierarchy (82–84). Moreover, anaerobic aromatic metabolism in *R. palustris* proceeds through the benzoyl-CoA pathway under photoheterotrophic conditions (3, 45, 46, 49, 85). Many lignin-derived catabolic routes generate condition-specific carboxylic acid or CoA-ester intermediates (61), positioning RPA2302 as a plausible activator of these metabolites for downstream assimilation under anaerobic stress. Although no single tier reconstructs a complete lignin-degradation pathway, the hierarchy recovers recognizable oxygen-dependent aromatic funneling nodes and anaerobic regulatory architectures while highlighting a substantial set of poorly annotated proteins whose context-specific recruitment remains underexplored in *R. palustris*.

### Growth-determinant proteins retain predictive power, rewire across oxygen regimes, and are underprioritized by pooled co-abundance analysis

Having identified a four-tier hierarchy of candidate growth-associated proteins, we asked three converging questions to evaluate whether this hierarchy captures a structured biological signal. Do these hub proteins relate to the rest of the proteome through static or condition-dependent relationships? Do they retain standalone predictive power? And would a conventional network approach have prioritized them? To address the first question, we measured how strongly each hub protein’s abundance correlated with every other protein in the data set, separately under aerobic and anaerobic conditions, keeping only strong and statistically reliable relationships (|*r*| ≥ 0.8, FDR < 0.05; Method S7; Fig. S4). Across all four tiers, the majority of hub co-abundance relationships were aerobic specific, consistent with the greater metabolic complexity engaged during growth on oxygen. This was most extreme for RPA3416, the highest-ranking tier 1 hub, which maintained 125 aerobic co-abundance partners and zero under anaerobic conditions. Yet within the same universal core, a counter pattern emerged: cysteine desulfurase NifS1 (RPA2463) formed 40 anaerobic-specific co-abundance relationships with no aerobic counterparts, and the chemotaxis receptor RPA3185 and uncharacterized protein RPA0477 were similarly anaerobic dominant. These proteins are universally important for prediction, but the proteomic neighborhood through which they are embedded is condition specific. Four tier 1 proteins—RPA2435, RPA4677, RPA4244, and RPA2671—formed no significant co-abundance relationships under either condition, indicating that their signal is not captured by strong pairwise coupling to the broader proteome. The rarity of significant co-abundance partners shared across both regimes precluded systematic assessment of sign reversals.

To ask whether the tiered proteins retain substantial standalone predictive power—rather than merely correlating with growth within the full model—we retrained the prediction model using each protein subset as the only input, under identical conditions (Method S9; Table S13). The 118-protein HH-consensus set preserved nearly all the predictive accuracy of the full 1,857-protein proteome (Spearman ρ = 0.745 versus ρ = 0.829), indicating that growth-rate information in the proteome is concentrated rather than diffuse. The 22 tier 1 universal core proteins alone—representing just 1.2% of all proteins measured—still predicted growth rate strongly across all conditions (ρ = 0.723). Tier 3 adaptive specialist proteins also performed well as a standalone set (ρ = 0.776), likely because their condition-specific recruitment is tightly tied to the substrate and oxygen combinations that most strongly differentiate growth rates. Tier 2 and tier 4 proteins were weaker in isolation (ρ = 0.54 and 0.42), consistent with roles that depend more strongly on the context of the full hierarchy.

The sharpest method benchmark came from a direct comparison with WGCNA, a standard method for identifying groups of proteins that move together and linking them to a biological outcome of interest. Applied to all 80 samples, WGCNA grouped the 1,857 proteins into 22 co-abundance modules, of which six were significantly associated with growth rate, together covering 910 proteins—nearly half the proteome (Method S8; Fig. S5A). The 22 proteins most central to these growth-associated modules were selected as WGCNA hubs and, as expected, each tracked growth rate strongly in its individual abundance. Yet when these 22 proteins were used as the sole input to the nonlinear prediction model, prediction failed (ρ = −0.42), while a simpler linear model on the same proteins performed reasonably well (ρ = 0.714). This dissociation is consistent with the interpretation that WGCNA hubs rise and fall with growth rate as part of a broad, coordinated response: they may function primarily as indicators of growth state rather than uniquely informative cross-condition determinants. The CorePredX-identified tier 1 proteins, by contrast, predicted growth equally well at the individual level (Fig. S5B; *P* = 1.00) yet were significantly less embedded in co-abundance modules than WGCNA hubs (Fig. S5C; *P* = 1.4 × 10⁻⁸), with proteins like RPA4677 and biotin carboxylase AccC (RPA2435) varying almost independently of the measured co-abundance network. These proteins may be underprioritized by network-based approaches despite ranking highly under dependence-aware predictive analysis. Together, these three lines of evidence support the view that the CorePredX hierarchy captures a dimension of growth-associated predictive structure that conventional co-abundance analysis does not readily access: while WGCNA identifies proteins that respond to changes in growth rate, the ML framework identifies a smaller set whose variation more directly and non-redundantly informs growth prediction.

## DISCUSSION

This study supports the interpretation that the metabolic versatility of *Rhodopseudomonas palustris* across lignin-derived aromatics is not achieved through wholesale rewiring of growth-associated biochemistry between environments, but instead through condition-specific regulatory and metabolic variation built around a shared predictive scaffold. Despite the pronounced oxygen-driven bifurcation of the abundance proteome—reflecting a genuine and well-characterized shift between respiratory and photoheterotrophic metabolic programs—a compact subset of proteins whose quantitative variation makes conditionally non-redundant contributions to growth prediction remains shared across regimes, even as many other growth-relevant features shift with oxygen context. Notwithstanding this proteome remodeling, growth prediction remained anchored to a substantially shared set of proteins across environments, consistent with a shared predictive scaffold supplemented by condition-specific extensions. Aerobic and anaerobic conditions, therefore, may represent alternative implementations of a shared growth-associated or regulatory architecture rather than fully distinct growth programs, an interpretation that emerges not from differential expression patterns alone but from the structure of model-derived feature importance across environments.

This interpretation is conceptually consistent with, and mechanistically extends, proteome allocation and bacterial growth-law frameworks developed primarily in model organisms. In *Escherichia coli*, a restricted set of proteome sectors—ribosomal proteins, metabolic enzymes, and stress-response components—collectively impose rate-limiting control across diverse growth conditions, with the relative size of each sector adjusting to balance translational capacity against metabolic demand (24, 86). Analogous sector-level constraints have been described in *Bacillus subtilis* (87–89) and in the metabolically versatile *Pseudomonas putida* (90–92), suggesting that sparse growth-rate control is a broadly conserved organizing principle of bacterial physiology rather than a peculiarity of fast-growing laboratory strains. The proposed universal core identified here in *R. palustris*—spanning translational capacity (TyrS, ArgE, and RpsD), coupled carbon storage and mobilization (PhbC-RPA4206), sulfur assimilation (CysA-NifS1), redox and iron homeostasis (Fur and RPA4244), anaplerotic biosynthesis (AccC and MutB), aromatic substrate commitment (RPA3761 and RPA3185), and an uncharacterized CBS-domain candidate (RPA3416)—is conceptually consistent with these conserved sectors while extending them into the domain of aromatic carbon catabolism and photoheterotrophic redox management unique to this organism’s lifestyle. The compact size of this core—22 proteins, fewer than 1.2% of the quantified proteome—and its ability to sustain independent growth-rate prediction (Spearman ρ = 0.723) together indicate that growth-associated predictive signal in *R. palustris* is concentrated rather than diffuse, consistent with emerging sparse-modularity principles in bacterial physiology (23).

Several specific findings within the proposed universal core have functional parallels in related organisms, suggesting that at least parts of this scaffold may reflect conserved growth-associated logic in metabolically versatile proteobacteria rather than being idiosyncratic to this experimental system. The co-occurrence of PhbC and RPA4206 as regime-invariant features is consistent with the established role of the PHB synthesis-mobilization cycle as a coupled redox and carbon buffer in purple non-sulfur bacteria, including *Rhodobacter sphaeroides* and *Rhodospirillum rubrum*, where PHB accumulation and remobilization are tightly coupled to growth rate under fluctuating carbon and light availability (30, 93). The co-identification of CysA and NifS1 as a sulfur-assimilation node parallels findings in *Synechocystis* and other phototrophs grown in minimal sulfur-limited media, where sulfate transport capacity and Fe-S cluster assembly have each been known to individually limit biosynthetic throughput; whether they jointly constitute a bottleneck in those organisms, as suggested here (37, 94–97), remains open. The regime-invariant contribution of the chemotaxis receptor RPA3185 is consistent with the demonstrated role of methyl-accepting chemotaxis proteins in *Azospirillum brasilense* and *Rhodobacter sphaeroides* in sensing aromatic acids and organic carbon sources. However, because the present experiments were performed in well-mixed liquid cultures (39, 98, 99), the mechanistic relevance of classical chemotaxis is unclear here, and any analogous role in *R. palustris* will require experimental follow-up.

A particularly notable implication of this hierarchy is the strong, regime-consistent predictive importance of RPA3416, an uncharacterized protein carrying two tandem CBS domains. CBS domain proteins are distributed across virtually all bacterial genomes and are widely proposed to function as adenylate energy sensors, coupling cellular energy charge to conformational regulation of associated proteins or complexes (42, 43). Despite their prevalence, standalone CBS-domain proteins remain functionally uncharacterized in most organisms, including well-studied model bacteria. The closest biochemically characterized analogs—CBS domain-containing family II inorganic pyrophosphatases in *Moorella thermoacetica* and *Desulfitobacterium hafniense*—use paired CBS domains to sense the ATP/AMP/ADP ratio and transduce energy charge directly into pyrophosphate flux regulation, coupling biosynthetic output to the adenylate energy state of the cell (44). The aerobic-dominant, substrate-invariant predictive contribution of RPA3416, combined with its maintenance of over 125 aerobic co-abundance relationships and complete absence of anaerobic connectivity, raises the specific and testable hypothesis that it may function as an energy-state-responsive regulator affecting biosynthetic or regulatory output during aerobic respiration on lignin-derived substrates—a metabolic context under which high respiratory flux may generate substantial adenylate dynamics and where energy charge fluctuations could plausibly influence growth throughput (100). Alternative explanations, including indirect transcriptional coupling to a true energy-sensing regulator, cannot be excluded without genetic dissection. Experimental validation through nucleotide-binding assays, co-immunoprecipitation to identify interacting partners, and deletion-strain growth phenotyping across the substrate panel remains essential before causality can be established.

A second key finding concerns the structural relationship between growth-rate constraint and co-abundance network connectivity. The WGCNA benchmark revealed a dissociation: proteins deeply embedded in growth-rate-associated co-abundance modules track growth rate equally well as the CorePredX-identified tier 1 proteins at the level of individual abundance correlation, yet fail to support nonlinear cross-condition growth prediction (ρ = −0.42 for the nonlinear model versus ρ = 0.714 for a simple linear model on the same proteins). This dissociation strongly suggests that growth-rate correlation and growth-rate constraint are not equivalent, and co-abundance connectivity may preferentially select proteins that participate in coordinated physiological responses to growth as reliable trackers rather than those whose variation uniquely encodes growth capacity. This distinction has a direct and underappreciated consequence for microbial systems biology: conventional network-based approaches, including co-expression and co-abundance analyses widely used to prioritize candidates in non-model organisms, may systematically underprioritize proteins like RPA4677—a protein with no experimentally characterized function in bacteria, carrying domains otherwise associated with eukaryotic cell death. Machinery that varies almost entirely independently of the rest of the proteome yet ranks among the most non-redundant predictive determinants identified here—precisely because autonomous variation weakens its status as a network hub.

This observation connects to a broader challenge in the systems biology of non-model organisms. For organisms like *R. palustris*, where genome annotation is incomplete and experimental characterization of individual proteins lags far behind sequence availability, the standard approach to building and gap-filling mechanistic models relies on BLAST-based homology transfer from well-characterized template organisms, phylogenetic inference, or domain-based functional assignment. These approaches are powerful for assigning putative catalytic functions to enzymes with recognizable active-site architectures, but are less well suited to capturing two classes of proteins that appear prominently in our growth-determinant hierarchy: first, proteins whose growth-relevant functions are mediated through regulatory, sensory, or scaffolding activities rather than catalysis, and for which sequence homology does not reliably predict physiological role in a new organism or metabolic context; and second, proteins like RPA3416 and RPA4677, whose domain architectures are recognizable in eukaryotic or distantly related bacterial contexts but whose specific biological functions remain uncharacterized even in well-studied organisms. Critically, even where homologs can be identified, functional redundancy among paralogs means that the most informative growth-associated member of a protein family cannot be determined from sequence alone—a problem that homology transfer does not resolve, and results in genome-scale models that may include the correct reaction but assign it to the wrong or incomplete set of gene-protein-reaction (GPR) associations. The consequence is that current mechanistic models of non-model organisms—including genome-scale metabolic and ME-model reconstructions—are likely to underrepresent the regulatory, sensory, and poorly annotated proteins that our analysis identifies as non-redundant predictive determinants associated with growth, not because these proteins are absent from the genome annotation, but because existing model-building pipelines have no principled basis for prioritizing them over their paralogs and homologs (101–104).

The four-tier hierarchy identified here suggests a potential route to addressing this gap in a targeted way. The 118 high-confidence determinants, and particularly the 22 universal core proteins, define a minimal, data-grounded set of growth-associated nodes whose experimental characterization and targeted inclusion in genome-scale reconstructions—whether through refined GPR assignments, regulatory constraints on flux bounds, or prioritization for biochemical characterization—could potentially inform model accuracy in ways that homology-based curation alone cannot achieve. More broadly, this approach suggests that cross-condition predictive modeling with dependence-aware interpretation could serve as a complementary front-end to mechanistic model curation in non-model organisms: rather than beginning exclusively from pathway completeness and filling gaps by homology, one could begin from the proteins that a generalizing growth model persistently requires for accurate prediction and use that experimentally prioritized set to guide both functional characterization efforts and GPR assignments. This represents a complementary philosophy of model building—anchored in predictive necessity rather than pathway reconstruction alone—and is particularly well suited to organisms whose annotation incompleteness and regulatory complexity limit the reliability of purely bottom-up approaches.

In conclusion, these results suggest a reorientation in how metabolic adaptability should be interpreted and engineered in versatile microorganisms. Rather than targeting individual pathway enzymes based on differential expression or pathway completeness, effective manipulation of growth on diverse carbon sources may benefit from identifying conserved candidate bottlenecks that persist across environments alongside the regulatory systems that gate access to them under specific substrate and redox conditions. The candidate growth scaffold identified here—spanning biosynthesis, redox balance, carbon storage, sulfur assimilation, chemosensory coupling, and energy-state sensing—highlights candidate control nodes for *R. palustris* that provide a starting point for both mechanistic investigation and model-guided engineering of metabolic versatility. The broader framework is readily extensible to other non-model organisms in which environmental plasticity and annotation incompleteness would otherwise obscure the conserved growth-associated structure encoded in the proteome.

### Limitations of the study

Several limitations of the present study warrant explicit consideration. SHAP-derived feature importance quantifies predictive necessity within the trained model and does not establish direct biochemical causality—experimental perturbation through deletion strains, biochemical characterization, and complementation will be required to determine whether the identified proteins are genuine growth-limiting bottlenecks or highly informative proxies. In addition, although 80 proteomic profiles were analyzed, the growth phenotype is defined at the level of 16 substrate-oxygen conditions, which limits the effective diversity of the response variable relative to the dimensionality of the proteomic feature space and may affect the stability of feature rankings. The proteomic measurements capture steady-state abundance at mid-exponential growth and do not resolve post-translational regulation, allostery, or metabolite-level constraints that may further shape growth control in ways invisible to abundance-based analysis. Pathway annotation for *R. palustris* CGA009 remains incomplete, and 64% of identified growth determinants lack definitive functional assignment—a gap that simultaneously motivates and is addressable by the experimental prioritization framework proposed here. Retaining acetate reference conditions in every training fold may also stabilize model calibration in ways that should be tested more directly through external validation or alternative partitioning schemes. Finally, the condition space explored, while chemically and redox-diverse, is finite; extending CorePredX to additional substrates, nutrient limitations, and temporal dynamics will be necessary to assess the generality of the inferred constraints and to determine whether the same candidate scaffold remains informative across a broader range of physiological perturbations.

## MATERIALS AND METHODS

### Growth experiments of *R. palustris* for different LBPs

*Rhodopseudomonas palustris* CGA009 (ATCC BAA-98) was used for all growth experiments. Strains were maintained at −80°C in glycerol stocks (20% [vol/vol] for *R. palustris*) and recovered on 112 Van Niel’s medium, supplemented with appropriate antibiotics (105). For growth assays, aerobic seed cultures of *R. palustris* were established and subsequently inoculated into photosynthetic medium (PM) (106) for LBP utilization experiments. Cultures were grown in 50 mL PM within 250 mL Erlenmeyer flasks under aerobic conditions or in 15 mL PM within sealed 16 mL Balch tubes under anaerobic conditions. Anaerobic cultures were illuminated continuously using an LED shelf light (SN-AG230-WIR-065) in an Algaetron incubator (Photon System Instruments). All cultures were maintained at 30°C with shaking at 275 rpm. PM was supplemented with 10 mM bicarbonate and 15.2 mM ammonium sulfate, and lignin-derived substrates were added at final concentrations of 1 mM as indicated. Where required, acetate (10 mM) was supplied to provide ATP and reducing equivalents necessary to initiate peripheral aromatic catabolism. To ensure that lignin breakdown products contributed directly to biomass formation rather than acting as inert co-substrates, cultures receiving both LBP and acetate were required to reach a final OD₆₆₀ exceeding that of acetate-only controls (Fig. S1). Growth curves represent the mean of three biological replicates.

### Proteome extraction, digestion, and LC–MS/MS

Cell pellets were lysed in Pierce RIPA buffer (Thermo Fisher Scientific) supplemented with 5 mM dithiothreitol and EDTA-free protease inhibitors (Roche) by heating at 95°C for 10 min with agitation. Lysates were clarified by centrifugation (16,000 × *g*, 15 min), and protein concentrations were measured using the CB-X protein assay (G-Biosciences). Fifty micrograms of protein per sample was alkylated with 20 mM iodoacetamide (40 min, dark), quenched with dithiothreitol, precipitated with acetone, and washed three times with 70% ethanol. Pellets were resuspended in 50 mM Tris-HCl (pH 8.0) and digested sequentially with Lys-C (4 h) and trypsin overnight at 37°C.

A pooled quality-control sample was generated by combining equal aliquots of all samples and injected after every 16 runs to monitor batch stability. The sample order was randomized by block randomization. Peptides were analyzed by nanoLC–MS/MS using an Ultimate 3000 RSLCnano system coupled to an Orbitrap Eclipse mass spectrometer (Thermo Fisher Scientific). Peptides were trapped on an Acclaim PepMap 100 column (75 µm × 2 cm) and separated on a C18 nano column (Peptide CSH, 75 µm × 250 mm) at 300 nL min⁻¹ using a 75-min gradient from 5% to 22% acetonitrile in 0.1% formic acid. Data were acquired in data-dependent mode with MS¹ scans at 120,000 resolution (m/z 375–1,500) followed by HCD MS² acquisition in the ion trap.

### Protein identification and preprocessing

Protein identification and quantification were performed using Proteome Discoverer v2.4 with Mascot against a combined *R. palustris* CGA009 UniProt database (UP000001426_258594) and a modified cRAP contaminant database. Searches assumed trypsin digestion with up to two missed cleavages, precursor tolerance of 15 ppm, and fragment tolerance of 0.06 Da. Carbamidomethylation of cysteine was set as a fixed modification, with methionine oxidation and asparagine/glutamine deamidation as variable modifications. Peptide-spectrum matches were filtered to 1% false discovery rate using Percolator. Only proteins identified by at least two unique peptides and five peptide-spectrum matches were retained.

Quantitative proteomics data were processed in MaxQuant Perseus. Proteins were required to be present in at least three replicates across all sample groups; others were discarded. LFQ protein intensities (our proxy abundances here) were log₂-transformed and missing values imputed using a downshift of 1.8 standard deviations and a width of 0.3 standard deviations. The resulting quantitative protein abundance profiles—comprising 80 proteomic profiles in total (5 biological replicates × 16 substrate–oxygen conditions: 8 substrates × 2 oxygen regimes), quantifying 1,857 proteins—provide the feature space for all downstream modeling. Of the 16 conditions, 2 acetate reference conditions (aerobic and anaerobic) were retained in every training fold to stabilize model calibration; the remaining 14 lignin-derived substrate–oxygen conditions were cycled through leave-one-condition-out evaluation to test generalizability. Unless otherwise specified, “16 conditions” refer to the full data set, and “14 conditions” refer to the set of held-out, non-anchor conditions. Quantile normalization was performed in R. Differential abundance between aerobic and anaerobic conditions was assessed on globally *z*-scored abundances using Welch’s two-sample *t*-test with Benjamini–Hochberg correction (FDR < 0.05), classifying proteins as aerobically or anaerobically enriched based on the sign of the mean difference.

### Model architecture, training, and cross-condition generalization

Proteomic data were analyzed using CorePredX, a learning and interpretive framework that maps condition-resolved proteome states to quantitative growth phenotypes while identifying features required for cross-environment generalization. The final data set comprised 80 proteomic profiles spanning 16 substrate-oxygen conditions, quantified across 1,857 proteins. Logistic growth-rate means and standard deviations were used as response variables, and condition metadata were retained to enforce condition-aware data partitioning.

Growth prediction employed a tapered multilayer perceptron (Decline-MLP) with three fully connected layers (311→120→46 units), ReLU activation, dropout (*P* = 0.1186), and the AdamW optimizer (learning rate 2.34 × 10⁻⁴; weight decay 1.47 × 10⁻⁶). Models were trained for up to 1,000 epochs with early stopping based on internal validation loss. A heteroscedastic Gaussian negative log-likelihood loss was used to account for replicate-level uncertainty in measured growth rates, jointly predicting mean μ(*x*) and variance σ²(*x*):


(1) $$L=\frac{1}{2}\sum_{i}\left[\frac{{\left(y_{i}-\mu_{i}\right)}^{2}}{\sigma_{i}^{2}}+log\;\sigma_{i}^{2}\right]$$

Generalization was evaluated using exhaustive leave-one-condition-out cross-validation. Each of the 14 environmental conditions was held out in turn, excluding all its replicates from training. Remaining conditions were split into internal training (~85%) and validation (~15%) sets. Two acetate reference conditions (Ac_ae and Ac_an) were included in every training fold to stabilize calibration because acetate provides stable, low-noise calibration anchors across oxygen regimes, enters central metabolism without requiring peripheral aromatic catabolism, and does not itself induce aromatic degradation pathways. This design limits fold-to-fold drift but also makes the evaluation partly dependent on the anchor conditions. Performance metrics (RMSE, Pearson, and Spearman correlations) were computed at both sample and condition-mean levels and summarized across folds. Model hyperparameters (learning rate, layer decay, dropout, weight decay, and initial hidden width) were optimized using Optuna with a TPE search (random seed = 42). Proteomic features were standardized to zero mean and unit variance prior to training.

### Monte Carlo SHAP analysis and growth-determinant analyses

Condition-resolved protein contributions to growth prediction were quantified using a Monte Carlo SHAP framework (see Method S1). Briefly, models were repeatedly retrained under leave-three-conditions-out cross-validation, in which all biological replicates from three non-anchor substrate–oxygen conditions were excluded per iteration, while acetate reference conditions (*Ac_ae* and *Ac_an*) were retained to stabilize calibration. Kernel SHAP was applied to held-out samples using training data as the background distribution, producing condition-resolved contribution profiles for all quantified proteins. These SHAP-derived importance profiles, rather than protein abundances, formed the basis for all downstream analyses.

Global growth determinants were identified by aggregating condition-resolved SHAP profiles across all environments and decomposing importance variance into regime-associated and condition-specific components (Method S2). Proteins were classified into aerobic, anaerobic, oxygen-bridging, or adaptive determinant classes based on global importance, regime variance, and aerobic–anaerobic effect size. HH analysis further refined this set using SHAP-based module construction and dependence-aware conditional perturbation to identify proteins whose quantitative variation was non-redundantly required for accurate growth prediction across environments (Methods S3 to S6).

### Simulation platform

All computational analyses for the CorePredX pipeline were conducted in Python (v3.10.18) using PyTorch (v2.4.1) with CUDA 11.8 and cuDNN 9.1, executed on the University of Nebraska–Lincoln’s Holland Computing Center SWAN cluster running Ubuntu Linux. Core models were trained on an NVIDIA Tesla V100S GPU (32 GB). The computational node contained dual Intel(R) Xeon(R) Gold 6248R CPUs (20 cores allocated per job) and 64 GiB of RAM, providing sufficient throughput for large-scale cross-condition learning, anchored LOOCV procedures, and SHAP-based interpretability analyses. This standardized environment enabled consistent benchmarking and reproducible execution of all CorePredX experiments.

## Data Availability

All the codes required to reproduce the results of the paper can be accessed at https://github.com/ssbio/CorePredX.

## SUPPLEMENTAL MATERIAL

Supplemental Material msystems.00383-26-s0001.docx Figures S1–S5 and Methods S1–S9.

Supplemental Tables msystems.00383-26-s0002.xlsx Tables S1–S13.

ASM does not own the copyrights to Supplemental Material that may be linked to, or accessed through, an article. The authors have granted ASM a non-exclusive, world-wide license to publish the Supplemental Material files. Please contact the corresponding author directly for reuse.
