# Supplementary material for: Machine learning reveals proteome-encoded growth determinants underlying metabolic versatility of Rhodopseudomonas palustris on lignin-derived aromatics
Source: mSystems. 2026 May 7;11(6):e00383-26. doi: 10.1128/msystems.00383-26 (PMC13289028; doi:10.1128/msystems.00383-26)
Supplement: Supplemental Material — Figures S1–S5 and Methods S1–S9. [file msystems.00383-26-s0001.docx]

# Supplementary Texts

*For:*

**Machine Learning Reveals Proteome-Encoded Growth Constraints Underlying Metabolic Versatility of *Rhodopseudomonas palustris* on Lignin-Derived Aromatics**

Abraham Osinuga^1^, Mark Kathol^1^, and Rajib Saha^1,†^

^1^Department of Chemical and Biomolecular Engineering, University of Nebraska-Lincoln, Lincoln, Nebraska, USA

^†^Email all correspondences to: [rsaha2@nebraska.edu](mailto:rsaha2@nebraska.edu)

**The file includes:**

[Supplementary Texts 1](#_Toc225427720)

[Supplementary Figures S1-S5 2-7](#_Toc225427721)

[Supplementary Methods S1 — Monte Carlo SHAP analysis for condition-resolved contribution profiles 10](#_Toc225427721)

[Supplementary Methods S2 — Global Determinant Analysis: pan-condition importance metrics and regime-variance decomposition 10](#_Toc225427722)

[Supplementary Methods S3 — High-Confidence Determinant (HH) analysis: module construction and dependence-aware conditional perturbation 11](#_Toc225427723)

[Supplementary Methods S4 —Protein-module construction based on SHAP importance profiles 11](#_Toc225427724)

[Supplementary Methods S5 — Module-level conditional perturbation analysis 12](#_Toc225427725)

[Supplementary Methods S6 — Feature-level conditional perturbation and redundancy classification (HH, HL, LH, LL) 12](#_Toc225427726)

[Supplementary Methods S7 — Hub co-abundance network construction and condition-resolved edge classification. 13](#_Toc225427727)

[Supplementary Methods S8 — WGCNA co-abundance network construction and growth-rate module identification. 14](#_Toc225427728)

[Supplementary Methods S9 — WGCNA co-abundance network construction and growth-rate module identification. 14](#_Toc225427729)

[References 15](#_Toc225427730)


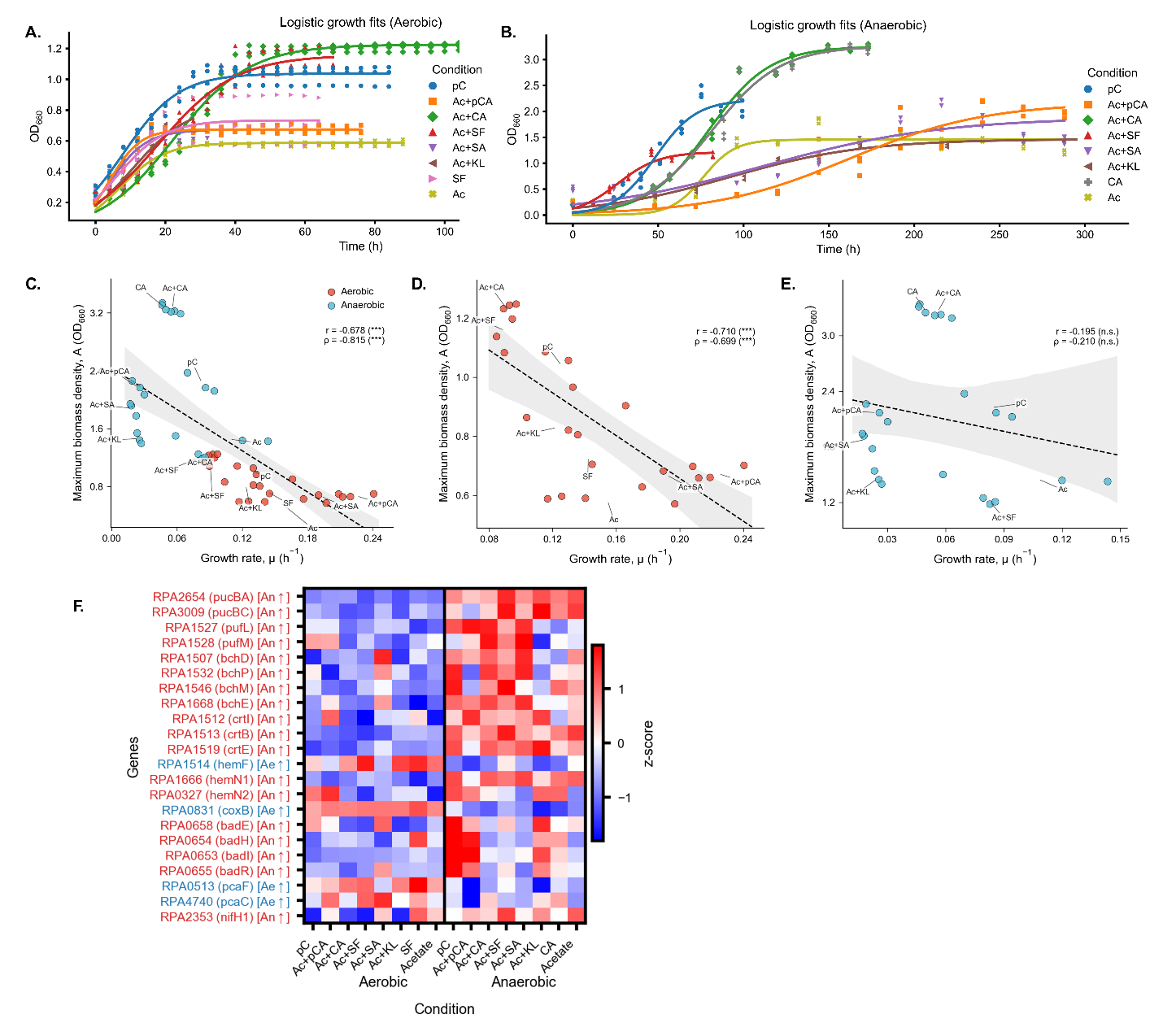


Figure S1. **Growth phenotypes and oxygen-dependent proteomic signatures during lignin breakdown product utilization in *Rhodopseudomonas palustris*.** **(A)** Logistic growth fits for aerobic cultures grown on lignin-derived substrates with or without acetate; points denote measured OD₆₆₀ values and lines indicate fitted models. **(B)** Corresponding growth dynamics under anaerobic, light-exposed conditions, revealing distinct substrate- and oxygen-dependent growth behaviors. **(C)** Inverse relationship between growth rate and maximum biomass density across all conditions. **(D)** Relationship restricted to aerobic cultures. **(E)** Absence of this relationship under anaerobic conditions, indicating that photoheterotrophic growth decouples the growth rate–yield trade-off characteristic of respiratory metabolism. **(F)** Heatmap of representative proteins showing oxygen-dependent proteomic reprogramming, including photosynthetic apparatus, bacteriochlorophyll biosynthesis, porphyrin metabolism, and aromatic catabolic pathways. Protein abundances are shown as row-wise z-scores, grouped by oxygen regime and substrate condition.


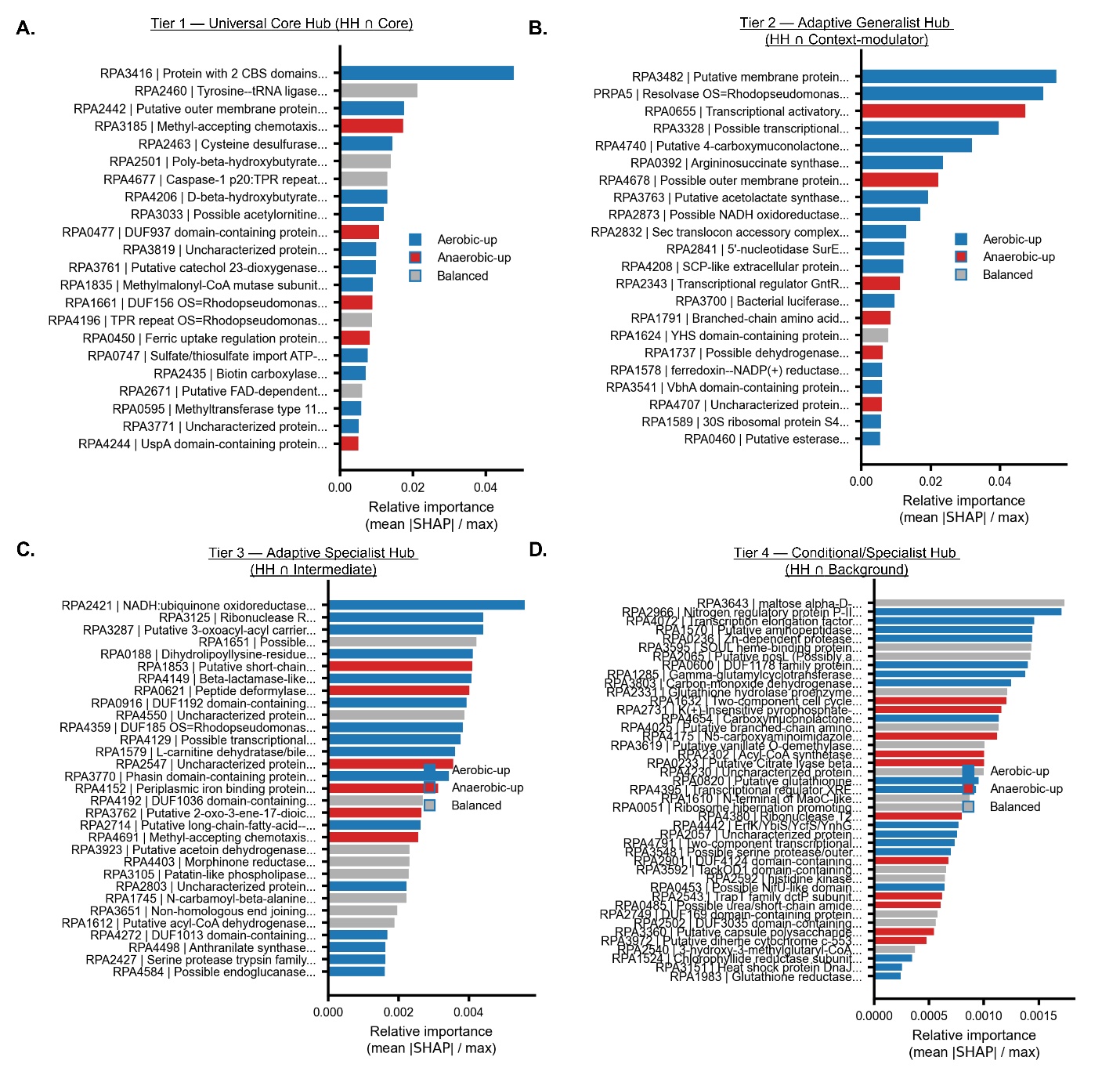
Figure S2. **Hierarchical organization of growth determinants identified by dependence-aware model interpretability.** **(A–D)** Ranked protein features within the four determinant tiers: **Tier 1**, Universal Core Hub; **Tier 2**, Adaptive Generalist Hub; **Tier 3**, Adaptive Specialist Hub; and **Tier 4**, Conditional/Specialist Hub. Bars indicate relative predictive importance (mean |SHAP| normalized to the global maximum). Colors indicate statistically significant differential protein abundance between oxygen regimes (aerobic-up, anaerobic-up; FDR < 0.05), or no significant difference (balanced).


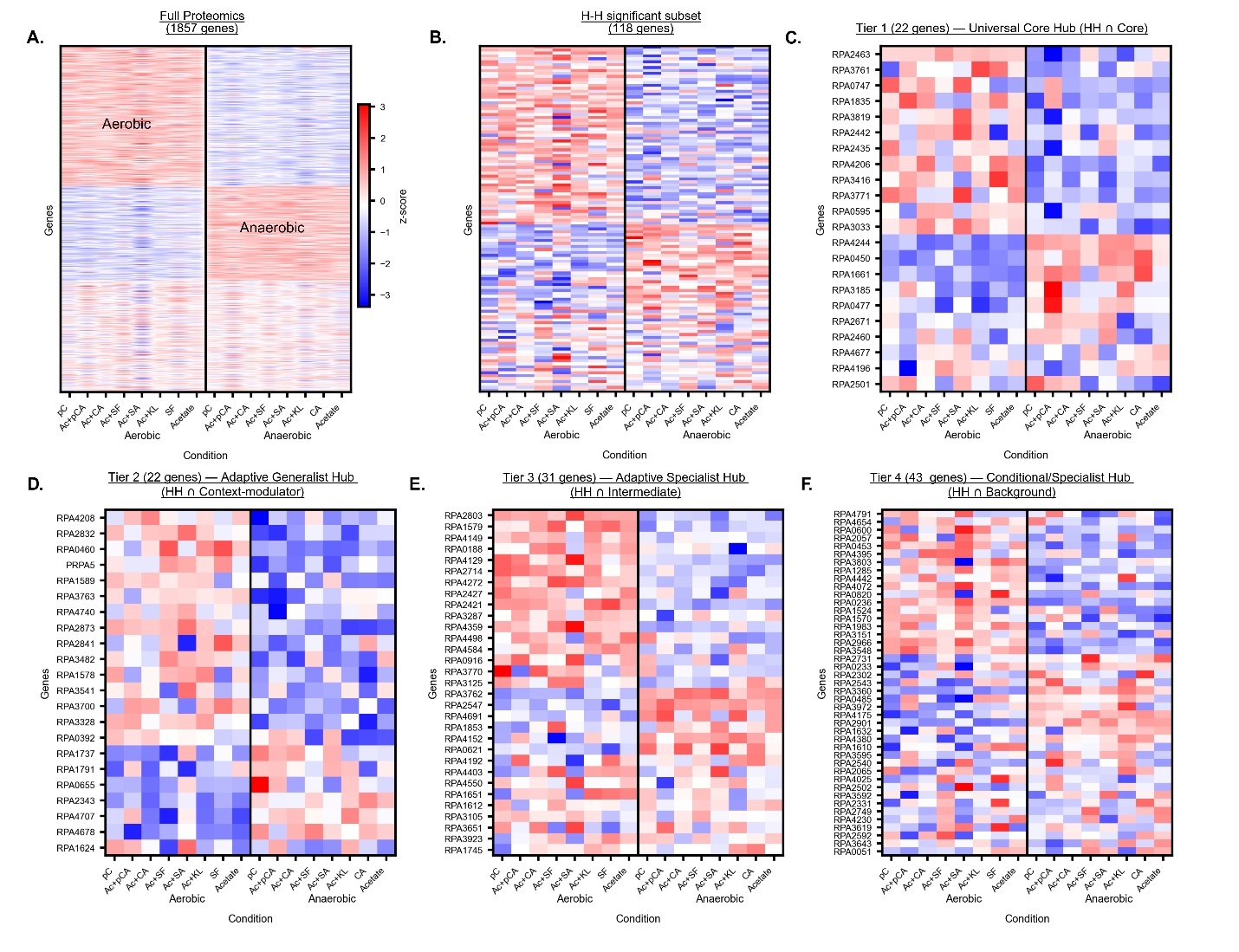
Figure S3. **Proteome-wide oxygen structuring and hierarchical refinement of growth determinants. (A)** Full proteome (1,857 proteins) shows strong oxygen-driven bifurcation in abundance space. **(B)** The HH-significant subset (118 proteins) retains oxygen structure while substantially reducing dimensionality. **(C–F)** Condition-resolved abundance patterns for proteins in the four determinant tiers: **Tier 1**, Universal Core Hub (22 proteins); **Tier 2**, Adaptive Generalist Hub (22 proteins); **Tier 3**, Adaptive Specialist Hub (31 proteins); and **Tier 4**, Conditional/Specialist Hub (43 proteins). Colors indicate z-scored protein abundance across substrate–oxygen conditions. These panels illustrate how strong oxygen-dependent proteomic remodeling coexists with a compact, hierarchically organized set of growth-relevant features identified through dependence-aware model interpretation.

**
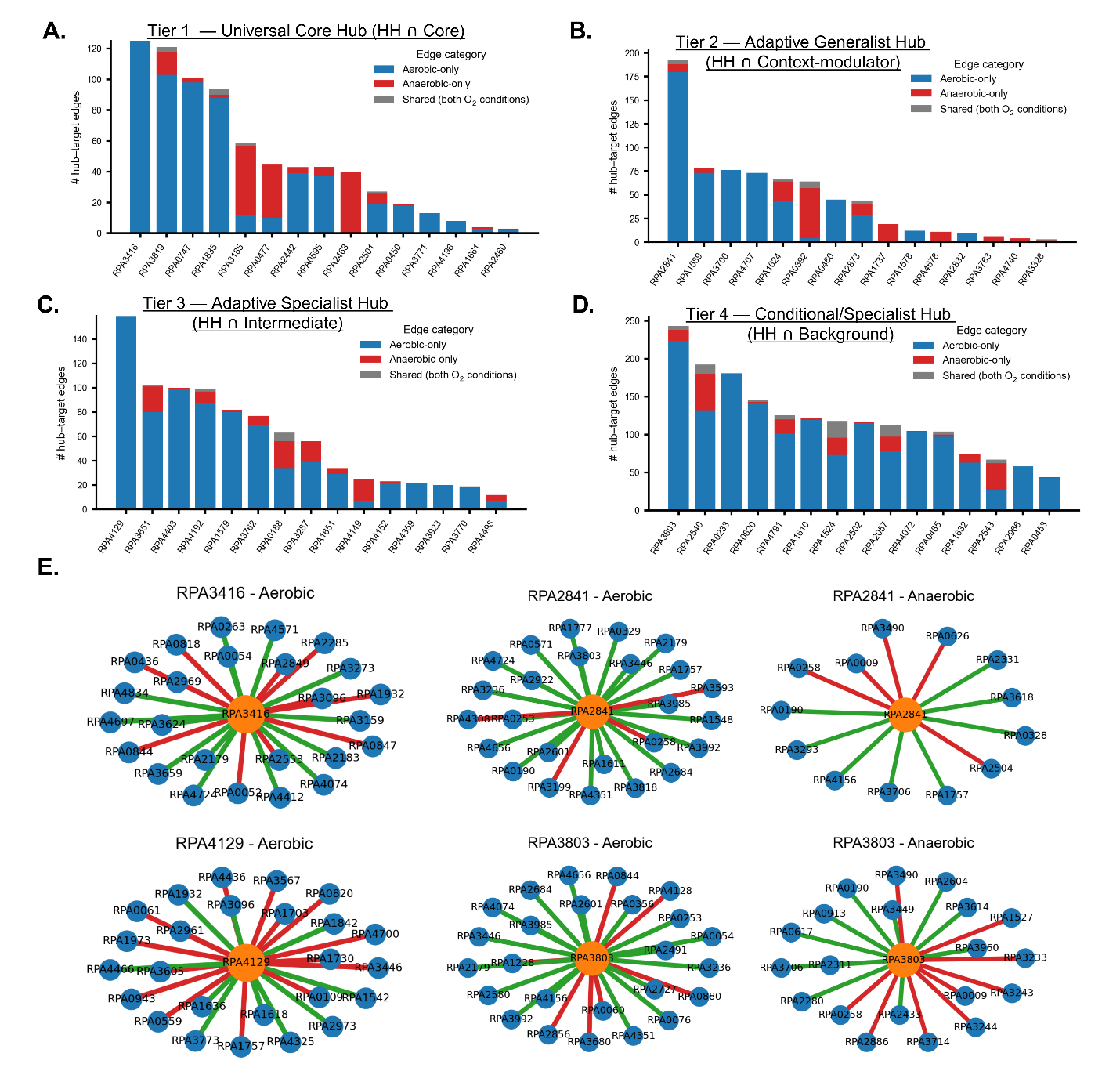
**

Figure S4. **Condition-resolved co-abundance hub networks across the four-tier growth determinant hierarchy.**  **(A–D)** Number of hub–target co-abundance edges per hub protein within Tier 1 **(A)**, Tier 2 **(B)**, Tier 3 **(C)**, and Tier 4 **(D)**, computed as pairwise Pearson correlations against all 1,857 quantified proteins stratified by oxygen condition and retained at BH-corrected q < 0.05 and |r| ≥ 0.8. Edge categories: aerobic-only (blue), anaerobic-only (red), shared with concordant sign (gray). **(E)** Star network visualizations for RPA3416 (Tier 1), RPA2841 (Tier 2), RPA4129 (Tier 3), and RPA3803 (Tier 4), showing the top 25 partners by |r|. Orange nodes: hub proteins; blue nodes: targets. Edge color indicates co-abundance sign (green: positive; red: negative); edge width scales with |r|. Aerobic and anaerobic panels shown for hubs with condition-dependent neighborhoods (RPA2841, RPA3803); aerobic only shown for RPA3416 and RPA4129, which form no significant anaerobic edges.


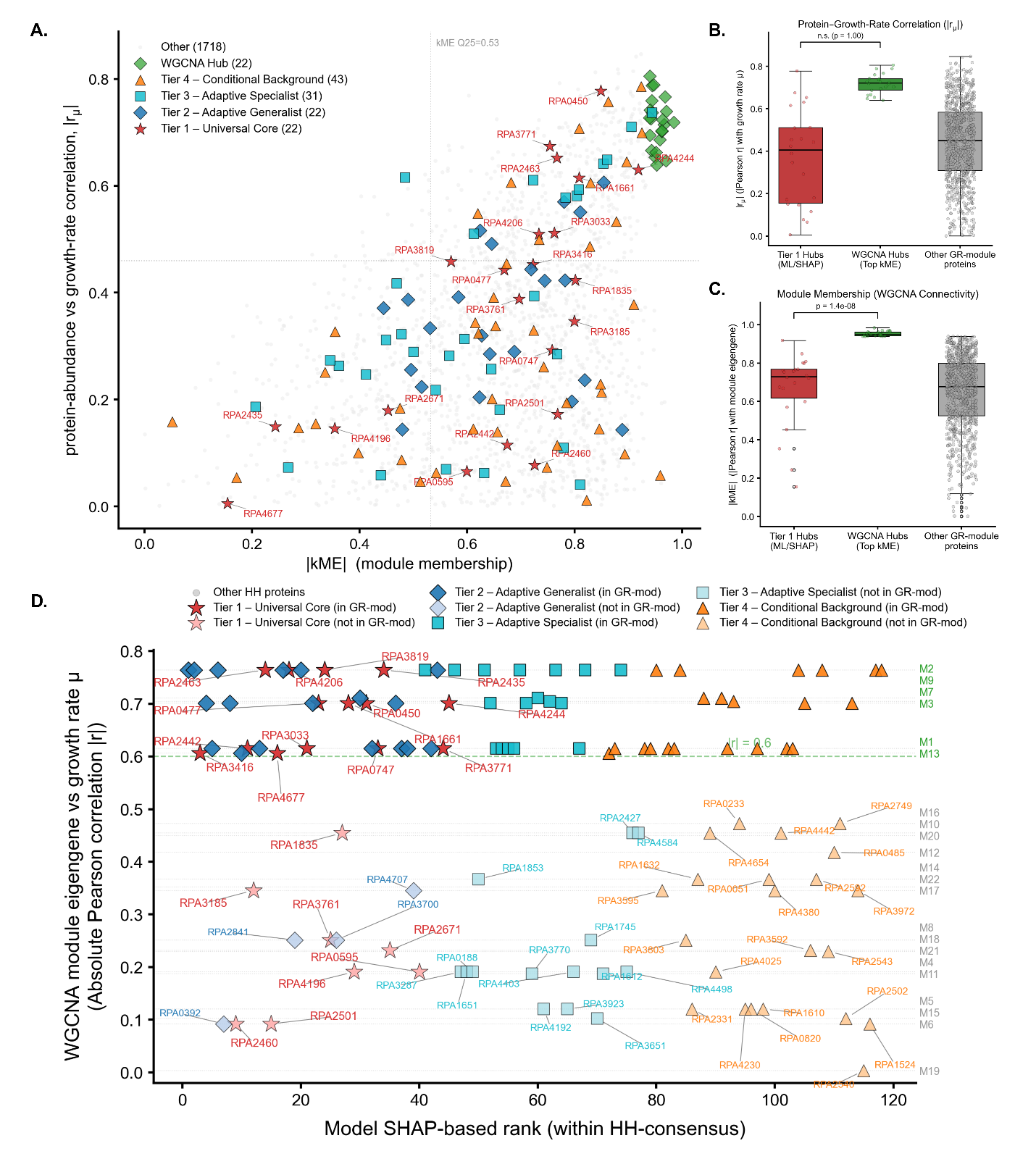


Figure S5. **CorePredX-identified growth determinants are as predictive as WGCNA hubs but systematically less connected within the co-abundance network.**  **(A)** All 1,857 proteins positioned by WGCNA module membership (|kME|; x-axis) and individual protein-abundance–growth-rate correlation (|r*_μ_*|; y-axis). WGCNA hubs (green diamonds) cluster in the upper-right; Tier 1 Universal Core proteins (red stars) are broadly distributed, with RPA4677 and RPA2435 among those showing low connectivity despite strong growth-rate association. Dashed vertical line: kME Q25 = 0.53. **(B)** Growth-rate correlation is statistically equivalent between Tier 1 and WGCNA hubs (Wilcoxon rank-sum, p = 1.00). **(C)** Module membership is significantly lower for Tier 1 proteins than WGCNA hubs (p = 1.4×10⁻⁸), demonstrating that ML-identified growth constraints would be missed by network analysis alone. **(D)** Model SHAP-based HH-consensus rank (x-axis) versus module eigengene–growth-rate correlation (y-axis) for all proteins, colored by tier and GR-module membership. Dashed line: |r| = 0.6 growth-rate module threshold; module labels on right. WGCNA procedures in Supplementary Methods S8.

**Supplementary Tables S1–S13** provide all supporting datasets for this study, including growth measurements, quantitative proteomics, model-derived determinant tiers, pathway annotations, and feature-reduction predictive ablation results for *Rhodopseudomonas palustris* CGA009 across lignin-derived substrates and oxygen regimes.

## Supplementary Methods S1 — Monte Carlo SHAP analysis for condition-resolved contribution profiles

To quantify condition‑resolved protein contributions, Monte Carlo SHAP analysis was performed. All non‑anchor conditions were enumerated, and every unique combination of three conditions was held out, yielding 364 leave‑three‑conditions‑out splits. For each split, the Decline‑MLP was retrained de novo, retaining the acetate anchors. Kernel SHAP was applied to held‑out samples using training samples as the background distribution, with 5,460 model evaluations per instance. SHAP values were computed for all proteins and aggregated across splits to obtain global and condition‑specific contribution profiles. These SHAP‑derived profiles, rather than protein abundances, were used for all downstream analyses.

To quantify how proteome composition contributes to growth-rate prediction across environmental contexts, we performed a Monte Carlo SHAP (SHapley Additive exPlanations) analysis prior to any clustering or module construction. SHAP decomposes model predictions into additive, sample-specific feature contributions, providing a principled measure of how individual proteins influence predicted growth rates independent of their absolute abundance. To rigorously probe extrapolative behavior, all non-anchor substrate–oxygen conditions were enumerated, and every unique combination of three conditions was treated as a held-out group, yielding 364 leave-three-conditions-out (L3CO) splits. In each split, all biological replicates from the selected conditions were excluded entirely from model fitting, while the two acetate reference conditions (Ac_ae and Ac_an) were retained in every training fold to stabilize calibration. The remaining conditions were partitioned at the condition level into internal training and validation sets, typically comprising eight training and three validation conditions per iteration, ensuring that no sample from a held-out condition influenced parameter estimation or early stopping.

For each L3CO split, the Decline-MLP was reinitialized and trained de novo using the same architecture, optimizer, and heteroscedastic loss formulation described above. Kernel SHAP was then applied to samples from the held-out conditions, using all samples from the corresponding training fold as the background distribution and test samples as instances to be explained. Each instance was evaluated under a fixed computational budget of 5,460 model calls, and SHAP values were computed for all 1,857 quantified proteins. This procedure yielded high-resolution, condition-aware estimates of how variation in individual protein abundances contributes to predicted growth rates, as learned by independently trained models.

Global contribution profiles were obtained by aggregating absolute SHAP values across all explained samples and Monte Carlo iterations and normalizing by the total number of evaluated instances. To preserve biological context, SHAP values were also averaged within each substrate–oxygen condition, producing a condition-by-protein matrix of mean absolute contributions. These condition-resolved SHAP profiles formed the foundation for all downstream analyses, including identification of regime-invariant versus context-dependent predictors, module discovery, and dependence-aware perturbation tests. Importantly, because these analyses operate on model-derived contribution profiles rather than on differential protein abundance, they isolate features that are repeatedly required for accurate growth prediction across environments rather than those that merely co-vary with specific conditions.

## Supplementary Methods S2 — Global Determinant Analysis: pan-condition importance metrics and regime-variance decomposition

To identify **global growth determinants**—proteins whose predictive influence persists across substrates and oxygen regimes—we consolidated condition-resolved SHAP perturbation results into a set of pan-condition importance metrics. Aggregating SHAP profiles across all 16 substrate–oxygen conditions yielded a condition-by-protein matrix in which each entry reflects the magnitude of the model’s reliance on a given protein within a specific environmental context. Because SHAP values are derived from independently trained models and therefore differ in scale, variance structure, and correlation geometry, they are not directly comparable across conditions. We therefore implemented an ANOVA-style variance decomposition framework to define global determinants based on model-derived importance structure rather than protein abundance.

Condition-wise SHAP magnitudes were first normalized by their respective median absolute values to enable comparability across heterogeneous substrates and oxygen regimes. From this normalized matrix, we computed for each protein (i) a global mean importance across all conditions, (ii) aerobic- and anaerobic-specific mean importance values, and (iii) their difference (ΔAn–Ae). Total importance variance was then partitioned into between-regime and within-regime components, yielding a regime variance fraction that quantifies the extent to which a protein’s predictive relevance is explained by oxygen availability rather than substrate-specific or idiosyncratic effects. Statistical significance of regime-associated biases was assessed using Welch’s t-tests comparing aerobic and anaerobic SHAP distributions for each protein, with multiple testing controlled by the Benjamini–Hochberg procedure (FDR < 0.05).

Proteins were subsequently classified using data-driven percentile thresholds applied to global mean importance, regime variance fraction, and aerobic–anaerobic effect size. High-importance proteins were defined as those exceeding the 75th percentile of global mean importance, whereas proteins below the median were designated background contributors. Among high-importance proteins, those exhibiting significant regime bias (q < 0.05) and high regime variance (top quartile) were classified as aerobic or anaerobic global determinants, depending on the sign of ΔAn–Ae. High-importance proteins with non-significant regime effects and low regime variance were classified as oxygen-bridging pan-condition global determinants, while remaining high-importance proteins were designated adaptive global modulators. Importantly, this Global Determinant Analysis operates entirely on SHAP-derived importance profiles and does not imply regime-invariant protein abundance, but rather identifies proteins whose predictive necessity for growth generalizes across environments.

## Supplementary Methods S3 — High-Confidence Determinant (HH) analysis: module construction and dependence-aware conditional perturbation

The High-Confidence Determinant (HH) analysis integrates module-level organization, conditional perturbation, and feature-level redundancy testing to identify proteins whose quantitative variation is non-redundantly required for accurate growth-rate prediction across environments. Whereas the Global Determinant Analysis identifies proteins with consistent predictive importance across conditions, the HH analysis refines this set by explicitly accounting for feature correlation, some form of constitutive pathway coupling, and shared regulatory structure. All steps in the HH analysis operate on condition-resolved SHAP importance profiles, not on protein abundance.

## Supplementary Methods S4 —Protein-module construction based on SHAP importance profiles

To organize the 1,857 quantified proteins into coherent analytical units, we clustered them according to the similarity of their condition-resolved SHAP importance profiles. For each protein, the Monte Carlo SHAP procedure produced a 16-dimensional signature summarizing its mean absolute attribution across all aerobic, anaerobic, and anchor conditions. These signatures were standardized and compared using cosine similarity to form a symmetric feature-by-feature similarity matrix. Affinity propagation clustering (damping = 0.80; preference equal to the median of off-diagonal similarities) was applied without pre-specifying the number of clusters, yielding 55 protein modules (1). Each protein was assigned a fixed module membership for all downstream analyses. All computations were performed with a fixed random seed (42) to ensure reproducibility.

## Supplementary Methods S5 — Module-level conditional perturbation analysis

To determine whether each module contributes non-redundantly to predictive performance, we evaluated its effect using a dependence-aware conditional perturbation framework applied across all Monte Carlo SHAP iterations. For a given module $c$, protein abundances $X_{c}$ were modeled as conditionally dependent on the remaining proteome $Z$ via ridge regression ($\alpha=1.0$) fitted exclusively on training conditions for that iteration. The fitted conditional mean was used to decompose module abundances into predictable, $\hat{X}_{c}$ and residual components, $R_{c}$:

$\hat{X}_{c}=\mathbb{E}[X_{c}\mid Z]$ (1)

$R_{c}=X_{c}-\hat{X}_{c}$ (2)

Within each held-out condition, only the residual component $R_{c}$was permuted, and perturbed inputs were reconstructed as:

$X_{c}^{(\pi)}=\hat{X}_{c}+\pi(R_{c})$ (3)

The Decline-MLP trained in that iteration was then evaluated on the perturbed test set, and the change in predictive error was recorded as:

$\Delta\mathrm{RMSE}_{c}=\mathrm{RMSE}\left( f\left( X_{c}^{\left( \pi\right)},Z \right) \right)-\mathrm{RMSE}\left( f\left( X \right) \right)$ (4)

Repeating this procedure across all Monte Carlo splits (with $n=364$ iterations) generated a distribution of $\Delta\mathrm{RMSE}_{c}$values for each module. For each module, we computed the mean effect $\mu$, standard deviation $\sigma$, and standard error:

$\mathrm{SE}=\sigma/\sqrt{n}$ (5)

Statistical significance was assessed using a one-sided Normal test of $H_{0}:\mu\leq0$, with Benjamini–Hochberg correction (FDR = 0.05). To exclude trivially small effects, we further required $\mu>2\cdot\mathrm{SE}$.

Only a minority of modules (13 of 55) produced a statistically significant increase in prediction error upon conditional perturbation (FDR < 0.05), identifying them as significant predictive modules rather than correlated background structure (Fig. 2C). Because growth-limiting constraints may be expressed through distinct proteomic configurations depending on substrate chemistry and oxygen availability, we further required that predictive structure be represented across all environmental contexts. Accordingly, from the full set of modules exhibiting positive perturbation effects (ΔRMSE > 0), we selected the top-ranking module for each substrate–oxygen condition. This procedure yielded a set of 13 constitutive predictive modules that collectively span all environments analyzed and served as the input for all subsequent feature-level conditional perturbation analyses (Fig. 2D).

## Supplementary Methods S6 — Feature-level conditional perturbation and redundancy classification (HH, HL, LH, LL)

After identifying statistically supported modules, we quantified individual protein contributions using a feature-level conditional perturbation framework analogous to the module-level analysis. For each selected module, every constituent protein $x_{j}$ was modeled as conditionally dependent on the remaining proteome $X_{(-j)}$using ridge regression fitted on training conditions only and per each Monte-Carlo iteration:

$x_{j}=\hat{x}_{j}(X_{(-j)})+r_{j}$ (6)

where $\hat{x}_{j}$ denotes the conditional mean and $r_{j}$ the residual. Within each held-out condition, only the residual component was permuted, yielding perturbed inputs $x_{j}^{(\pi)}$, while retaining the measured covariance structure of the full proteome:

$x_{j}^{(\pi)}=\hat{x}_{j}+\pi\left( r_{j} \right)$ (7)

The trained Decline-MLP was evaluated on the perturbed test set, and the resulting change in prediction error was computed as:

$\Delta\mathrm{RMSE}_{j}=\mathrm{RMSE}(f(x_{j}^{(\pi)},X_{-j}))-\mathrm{RMSE}(f(X))$ (8)

Repeating this procedure across all Monte Carlo iterations generated a distribution of $\Delta\mathrm{RMSE}_{j}$ values for each protein. For each protein, we computed the Monte Carlo mean effect size $\mu_{j}$ of $\Delta\mathrm{RMSE}_{j}$, standard deviation $\sigma_{j}$, and standard error. A one-sided Normal test of $H_{0}:\mu_{j}\leq0$ was performed using the z-statistic $z_{j}=\mu_{j}/\mathrm{SE}_{j}$, and p-values were adjusted using the Benjamini–Hochberg procedure (FDR = 0.05). To ensure that selected proteins exhibited both statistical support and meaningful effect size, we imposed a joint criterion requiring $q_{j}<0.05$ and $\mu_{j}>2\text{ }\mathrm{SE}_{j}$. Proteins passing both filters were designated as feature-level contributors within their respective modules.

To differentiate proteins whose contributions were robust to the choice of conditioning set from those whose importance depended on local intra-module structure, we repeated the above analysis using two conditioning definitions: (i) conditioning on all proteins outside the focal module, and (ii) conditioning on all other proteins in the dataset. The resulting pair of effect estimates for each protein was subsequently used to classify features into high–high (HH), high–low (HL), low–high (LH), or low–low (LL) importance quadrants. This dual-conditioning procedure provided a reproducible and model-agnostic measure of feature-level contribution that captures both unique and redundancy-mediated predictive structure within the proteome.

## Supplementary Methods S7 — Hub co-abundance network construction and condition-resolved edge classification.

To test whether hub protein co-abundance neighborhoods are static or reorganize between oxygen regimes, pairwise Pearson correlations were computed between each hub protein and all 1,857 quantified proteins, stratified by oxygen condition (Aerobic: 40 samples; Anaerobic: 40 samples). Correlations were computed independently for each hub–target pair within each condition using only samples with finite values for both proteins (minimum 5 valid observations required). All p-values within each tier–condition combination were corrected for multiple testing using the Benjamini–Hochberg procedure, and an edge was retained only at FDR-corrected q < 0.05 and |r| ≥ 0.8, ensuring only strong and reproducible co-abundance relationships were considered. This yielded 80,740; 80,740; 113,212; and 156,004 pairwise tests per tier per condition for Tiers 1–4 respectively. Each hub–target pair was then classified into one of four categories: aerobic-only (significant under aerobic conditions only), anaerobic-only (significant under anaerobic conditions only), shared with concordant sign (significant in both conditions with the same directionality), or sign-reversed (significant in both conditions but with opposite directionality). No degree cutoff was imposed; partner count was determined entirely by the data. For network visualization, each hub was displayed alongside its top 25 co-abundance partners ranked by |r| within each oxygen condition. Edge color encodes the sign of the co-abundance relationship within each condition (green: positive; red: negative) and edge width is scaled proportionally to |r| (Fig. S4).

## Supplementary Methods S8 — WGCNA co-abundance network construction and growth-rate module identification.

A signed weighted protein co-abundance network was constructed from the full 80-sample × 1,857-protein LFQ abundance matrix using the Weighted Gene Co-expression Network Analysis (WGCNA) framework (2). A signed adjacency matrix was computed as:

$a_{ij}=[(1+r_{ij})/2]^{\beta}$, (9)

where $r_{ij}$is the Pearson correlation between proteins $i$and $j$and $\beta=2$is the soft-thresholding power. Adjacency values were transformed into a Topological Overlap Matrix (TOM) to capture shared neighborhood structure, and modules were identified by Affinity Propagation clustering on the TOM similarity matrix (1) using a precomputed affinity kernel, allowing the number of modules to be determined by the data without imposing an arbitrary cluster count. This procedure converged in 42 iterations yielding 22 modules ranging from 20 to 301 proteins. Module eigengenes were computed as the first principal component of the standardized within-module abundance matrix and correlated with per-sample growth rate using Pearson correlation; significance was assessed by Benjamini–Hochberg FDR correction across all 22 modules. Modules achieving |r| ≥ 0.6 at FDR < 0.05 were designated growth-rate-associated, yielding six modules (M1–M3, M7, M9, M13) encompassing 910 proteins. Module membership (kME) for each protein was defined as the absolute Pearson correlation between its abundance profile and its assigned module eigengene. WGCNA hub proteins were defined as the top 22 proteins by kME within growth-rate-associated modules, matched in number to the Tier 1 Universal Core set for controlled comparison. Group differences in individual protein-abundance–growth-rate correlation and kME were assessed by two-sided Wilcoxon rank-sum tests (Supplementary Fig. S5B–C).

## Supplementary Methods S9 — WGCNA co-abundance network construction and growth-rate module identification.

To test whether each protein subset independently encodes sufficient information to predict growth rate, the Decline-MLP prediction model and a linear ElasticNet baseline were each retrained and evaluated using the indicated protein subset as the sole input, under identical condition-anchored leave-one-condition-out cross-validation. Hyperparameters were fixed at values optimized on the full proteome (n_layers = 3, decay = 0.388, dropout = 0.119, learning rate = 2.34×10⁻⁴, weight decay = 1.48×10⁻⁶) to ensure performance differences across subsets reflect information content rather than architectural tuning. For each subset, the hidden layer width was selected by grid search over a range scaled proportionally to the number of input features, with the best configuration chosen by Spearman correlation on held-out predictions. Performance was evaluated as Spearman ρ, Pearson r, and RMSE across all held-out predictions. Results are reported in Supplementary Table S13.

# References

1. Frey BJ, Dueck D. 2007. Clustering by passing messages between data points. Science 315:972–976.

2. Langfelder P, Horvath S. 2008. WGCNA: an R package for weighted correlation network analysis. BMC Bioinformatics 9:559.
